# Submovements in manual tracking: people with Parkinson’s disease produce more submovements than age-matched controls

**DOI:** 10.1186/s12984-025-01592-1

**Published:** 2025-03-06

**Authors:** Lior Noy, Sharon Hassin-Baer, Tsvia Fay-Karmon, Noora Kattouf, Simon Israeli-Korn, Robrecht van der Wel, Jason Friedman

**Affiliations:** 1 Faculty of Business Administration, Ono Academic College, Kiryat Ono, Israel; 2 Movement Disorders Institute, Department of Neurology, Chaim Sheba Medical Center, Ramat Gan, Israel; 3 Faculty of Medical & Health Sciences, Tel Aviv University, Tel-Aviv, Israel; 4 Department Physical Therapy, Stanlyer Steyer School of Health Professions, Faculty of Medical and Health Sciences, Tel Aviv University, Ramat-Aviv, Tel-Aviv, 6997801 Israel; 5 Sagol School of Neuroscience, Tel Aviv University, Tel-Aviv, Israel; 6 Rutgers University, Camden, NJ USA

**Keywords:** Parkinson’s disease, Tracking, Slow movements, Smoothness, Submovements

## Abstract

**Background:**

In general, people are unable to produce slow, smooth movements - as movements become slower (i.e., with longer durations), they become jerkier. A hallmark feature of Parkinson’s disease is bradykinesia - slowness of movement. In this study, we investigate the intersection of these two observations - how do people with Parkinson’s disease (PwP) perform in a slow tracking task, and how does it vary as a function of movement frequency? On the one hand, as PwP move more slowly in day-to-day life, they may be better in a slow tracking task. On the other hand, their general impairment in movement production may lead to worse tracking outcomes.

**Methods:**

We used a well-tested tracking task known as the one-person mirror game, where participants control the left-right movement of an ellipse on a graphics tablet. They did so using a stylus and were instructed to match the horizontal location of a stimulus, an ellipse moving in a sinusoidal fashion at different movement frequencies and peak velocities. We calculated the submovement rate, identifying both type 2 (acceleration zero crossings) and type 3 (jerk zero crossings) from the trajectories, as well as relative position error (dX) and mean timing error (dT). To account for age-related performance decline, we tested three groups: PwP (*N* = 31), age-matched controls (OC; *N* = 29), and younger controls (YC; *N* = 30) in a cross-sectional study, and used mixed-design ANOVAs to compare across groups and movement frequencies.

**Results:**

We reproduced earlier results showing that slow movements (i.e., with lower frequencies) require more submovements to track. PwP also generally performed more submovements than the other two groups, but only for type 3 submovements, whereas OC and YC performed submovements at a similar rate. Younger controls (YC) performed fewer tracking errors than older participants (both PwP and OC), and OC performed better than PwP.

**Conclusions:**

The ability to smoothly track showed an age-related decline, with PwP producing more errors and using more submovements. This may be due to reduced automaticity in movement production. The findings of the study can be used to guide optimal movement frequencies for motor training for older adults and PwP.

**Supplementary Information:**

The online version contains supplementary material available at 10.1186/s12984-025-01592-1.

## Background

An integral aspect of healthy human movement is smoothness [1–3] - movements produced continually and without interruptions [1]. Movements may be smooth due to the intrinsic properties of a single muscle [4], or the coordinated action of multiple muscles [3]. However, there is a breakdown of smoothness in long-duration strokes (that is, movements from start-to-stop, at lower temporal frequencies). Such long-duration strokes are not smooth. Rather, they are divided into several submovements [5–8]. Submovements are typically defined as discrete movements that can be combined to make a larger movement [9]. The mechanism underlying these breakdowns is not well-understood, and this phenomenon is probably the result of several factors. These factors might include: a lack of previous experience with slow movements (we usually do not need to move very slowly, but see [8] for an interesting counter-example); factors relating to visuomotor transformations, e.g. how visual feedback is used [10]; biomechanical factors, e.g. how far the movement frequency is from the resonant frequency of the limb [11]; and possibly, an inherent limitation in the nervous system, e.g. a limited range of submovement durations observed in neural correlates [12, 13]. We note that we have moved here from talking about ‘long duration’ to ‘slow’. This is correct only given a constant distance (the length of the tracked linear motion in our study). Otherwise, it is, of course, possible to move with different speeds (slower or faster) using long duration strokes, depending on the traveled trajectory.

**Fig. 1:**
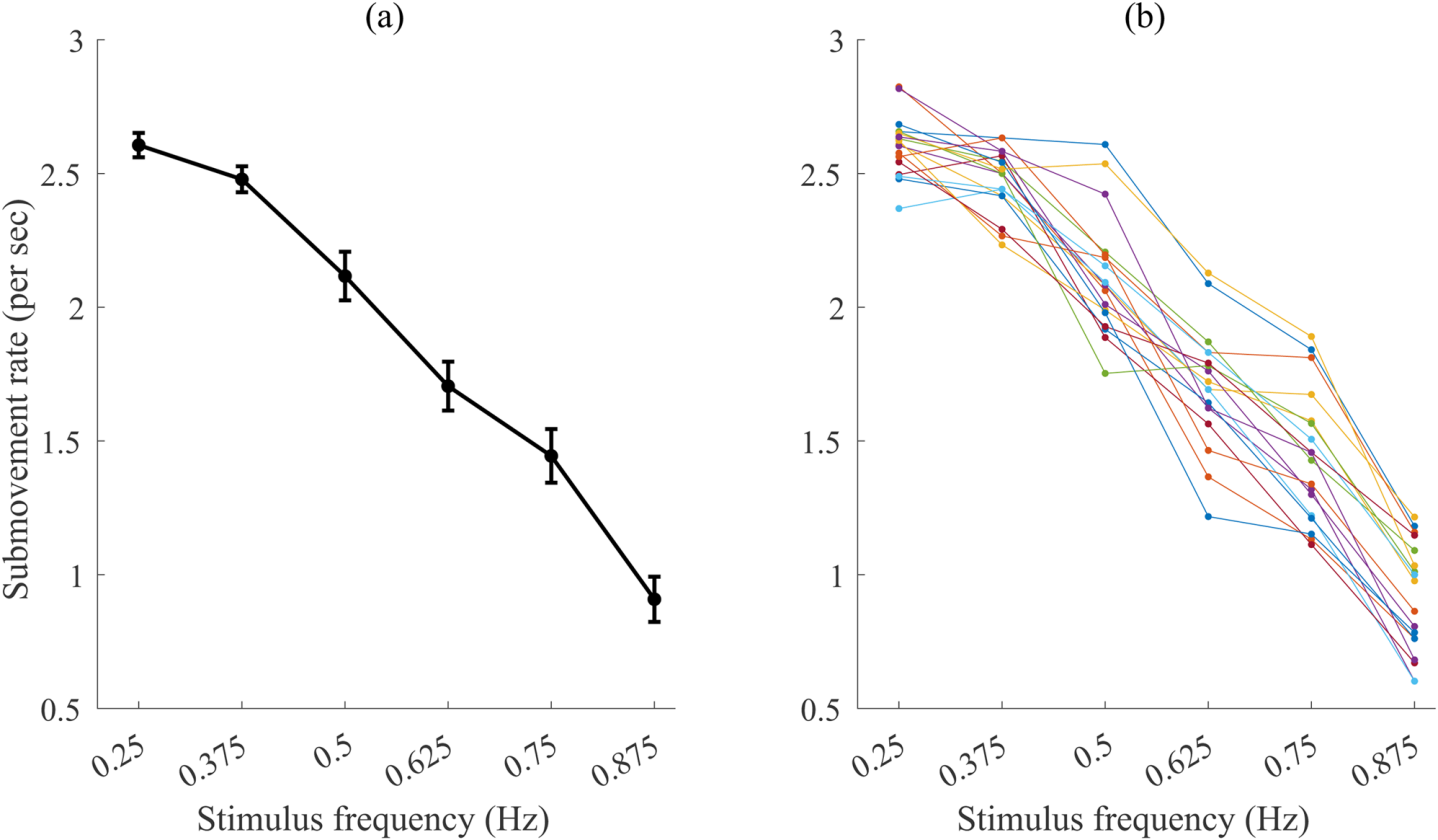
Consistent relationship of submovement rate and stimulus frequency in manual tracking. Submovement rate as a function of stimulus frequency, from the data in our previous study [6], with the data presented as (**a**) mean and standard error over all participants, and (**b**) individual participants (*N* = 18). Submovement rate here combines both type 2 and 3 submovements (see methods section)

Regardless of the mechanism, the inability of people to perform slow and smooth strokes was reported in several studies using different experimental setups, including point-to-point movements [14], interception of a moving target [15], paced movements [5], and manual tracking of rhythmic motions [6, 16]. The evidence is consistent: as movement frequency decreases, smoothness is lost, and longer-duration movements are divided into several submovements. In this study, we define submovements based on acceleration and jerk zero-crossings [14]. For example, using a simple manual tracking setup, we observed this pattern - a higher rate of submovements at lower frequencies - in several studies [6, 17]. Moreover, this pattern is observed not only at the group level but also for individual participants (see Fig. 1). We note that while in some cases submovements are related to accuracy, where submovements perform a corrective role, in other cases, submovements occur due to other factors [14]. Specifically, ample evidence suggests that submovements appear to be inevitable at low stimulus frequencies [6].

Our goal in the current paper is to study this general pattern - an increase in submovement rates for slower movements - in people with Parkinson’s disease (PwP). We suggest that studying PwP is an interesting case study for understanding the planning and execution of submovements, in particular for slower movements. On the one hand, PwP have difficulties with many aspects of their movement, including movement planning, initiation, and execution [18–20]. That is, we can hypothesize that PwP will produce more submovements than healthy controls across different movement frequencies, which will result in a constant upward shift of the submovement/frequency curve of Fig. 1. Several studies [19, 21–23] report a higher rate of submovements of PwP in reaching tasks (and see below). A more nuanced possibility is that PwP perform more submovements than healthy controls only in a certain range of movement frequencies. As we see that slow and smooth strokes are difficult in general, maybe PwP will have even more difficulties with these movements than healthy participants.

On the other hand, PwP move more slowly. Bradykinesia (from Greek: *brady* = low; *kinesis* = movement), the tendency to move very slowly, is a hallmark feature of Parkinson’s disease (PD) [24]. It is, therefore, possible that PwP have gained more experience with slow movements, and are better than healthy controls in their ability to perform slow and smooth strokes. The main goal of this research is to rigorously measure the rate of submovements in PwP and healthy controls across a range of frequencies to contrast these two options.

Parkinson’s disease is a heterogeneous disorder with multiple subtypes, with the most common subtypes being tremor-dominant, and postural instability and gait difficulty [25]; other less common subtypes are also observed. As a consequence of this, we expect varied performance on motor tasks when examining PwP due to these different subtypes. It is important to differentiate between submovements and tremor here; submovements are composed of lower frequencies and longer durations than tremor, which is known to be amplified in PwP. The question here is whether the rate of submovements also differs between healthy controls and PwP. We also note that there is evidence that tremor intensity in Parkinson’s has an effect on movement initiation but not movement time in a reaching task [26]. As we are using a continuous tracking task, we expect that tremor will have less of an effect (compared to point-to-point tasks). Similar to the approach of [26], we will filter the data to reduce the effect of tremor on the outcome measures.

Previous studies have suggested that PwP might produce more submovements than age-matched controls, at least for some types of submovements [19, 21–23]. These results are not conclusive, however. Moreover, the aforementioned studies employ point-to-point reaching tasks, where participants choose their own movement trajectories and timing. It is possible that PwP do not perform more submovements in general, and that the higher number of submovements reported in these studies is the result of an experimental confound: for example, as PwP move more slowly in general, they perform slower reaching movements, and this leads them to produce more submovements. The observation of Dounskaia et al. [21] reporting that PwP move more slowly in their reaching task (Fig. 2 from [21], lower peak velocity for PwP vs. controls) supports this interpretation.

**Fig. 2:**
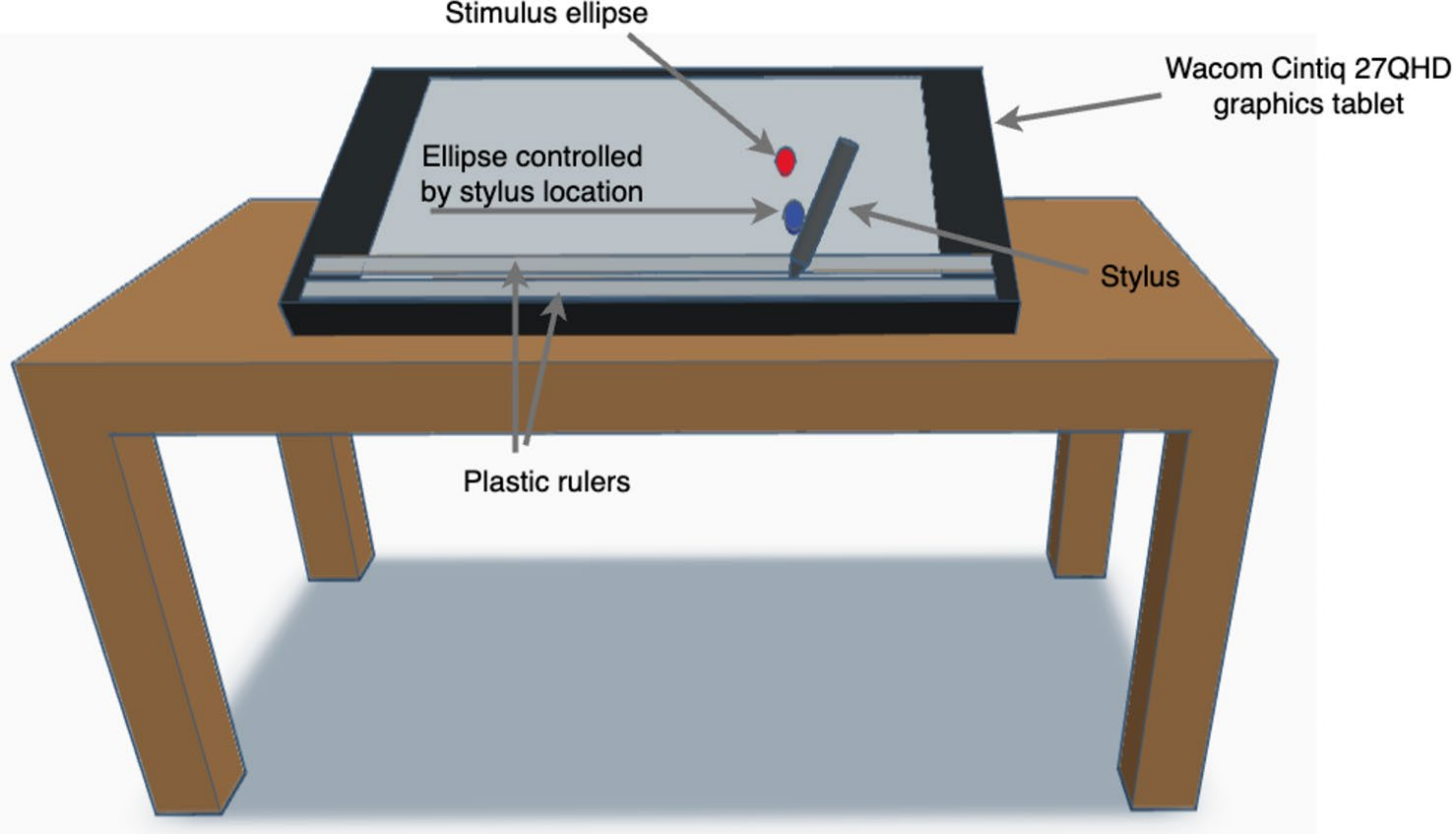
Experimental setup. The participants moved a stylus back-and-forth to the left and right (in a channel composed of two rulers) to control the horizontal position of the lower blue ellipse. They were instructed to track the horizontal position of the upper red ellipse, which oscillated for 18–22 s at a constant amplitude and frequency. 35 segments at different frequencies and amplitudes were used in a counterbalanced way over 11 one-minute trials

Here, we aimed to overcome this possible confound by employing a tracking task. As movement speeds and durations are held constant in tracking tasks, different participants will be guided to perform movements with the same frequencies and amplitudes, allowing us to better compare the movements of PwP and healthy controls [27].

**Fig. 3:**
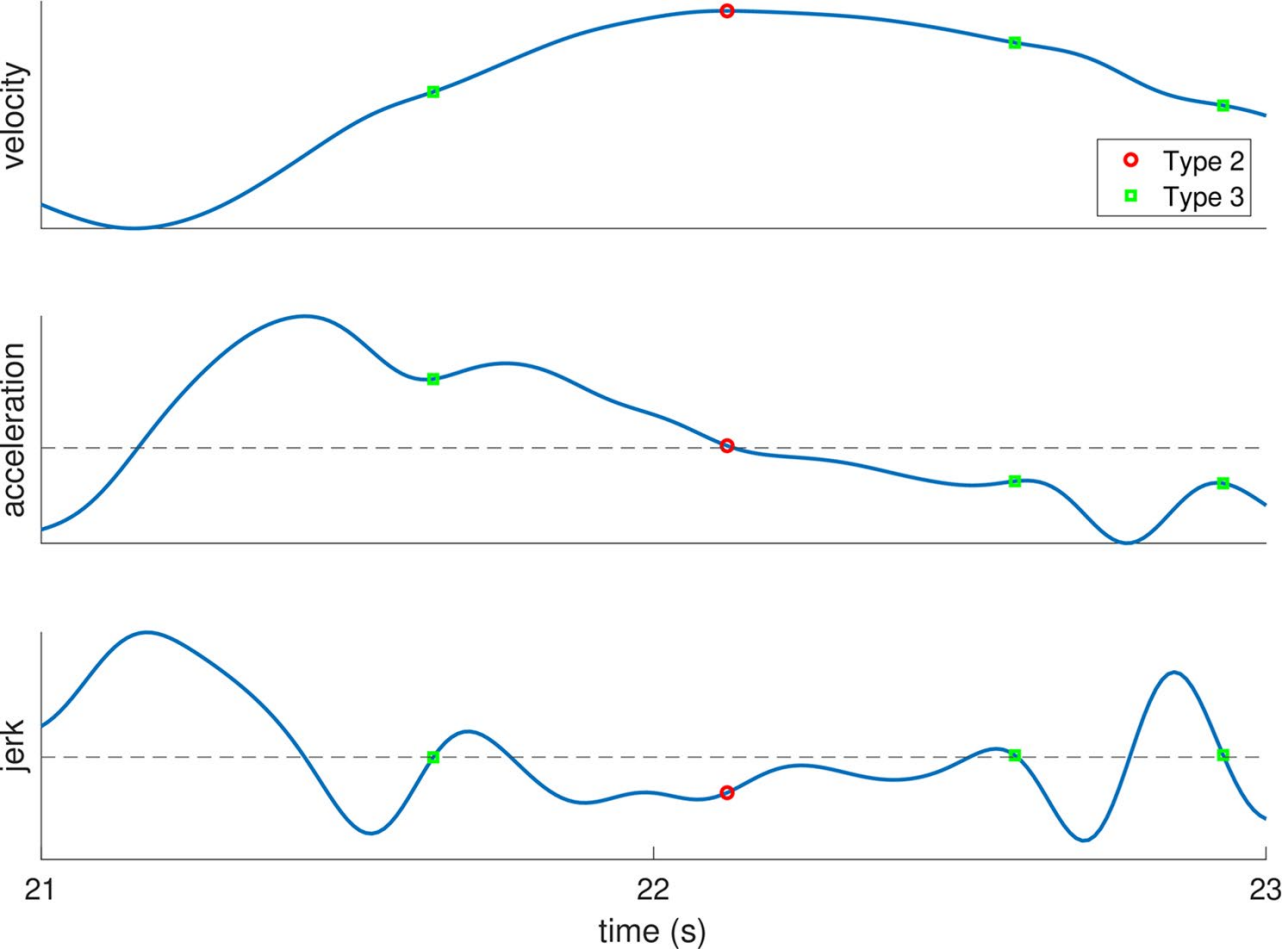
Examples from a typical trajectory showing how type 2 and type 3 submovements are defined. The three graphs show the velocity, acceleration, and jerk of the same time points. The dashed line indicates zero. Type 2 submovements (red circles) correspond to peaks in the velocity profile (zero crossings from positive to negative in the acceleration). Type 3 submovements (green squares) correspond to inflection points in the velocity profile - defined as jerk zero crossings from negative to positive when the acceleration is positive, or jerk zero crossings from positive to negative when the acceleration is negative

We used a particular manual task, the one-person mirror game that was used in previous studies ([6, 17] and see Fig. 2). We asked PwP and healthy controls to accurately track linear motions at different frequencies and amplitudes, using a similar sample of motions as in previous studies (see Table 1 in [6]), but used slower movement speeds to make the task manageable for these populations. We measured submovement rate and movement errors (relative spatial errors (dX) and timing errors (dT) [6, 17]). In addition, following Dounskaia et al. [14, 21], we measured submovements using a more general definition than we have used before. Specifically, we used two different measures for detecting submovements (see Fig. 3). The first measure is similar to what we used before, by detecting submovements based on acceleration zero crossings (AZC, termed type 2 submovements in [14, 21]). Our second measure detects submovements based on jerk zero crossings (JZC, termed type 3 submovements in [14, 21]). We use the terminology of type 2 and type 3 submovements hereafter. This addition might be important: in a previous study, there were no significant differences between PwP and controls in the rate of type 2 submovements, while PwP performed *more* type 3 submovements (which are more common, see Fig. 4 in [21]) than control participants in some tasks [21]. While several previous studies have measured continuous tracking in PwP [28–31], these studies did not report submovement analyses. To the best of our knowledge, the only publication analyzing submovements in continuous tracking in PwP is a conference paper with 3 participants, comparing performance in the “on” versus “off” medication states [32]. Here, we will analyze both type 2 and 3 submovements to compare performance across a range of different movement frequencies.

**Fig. 4:**
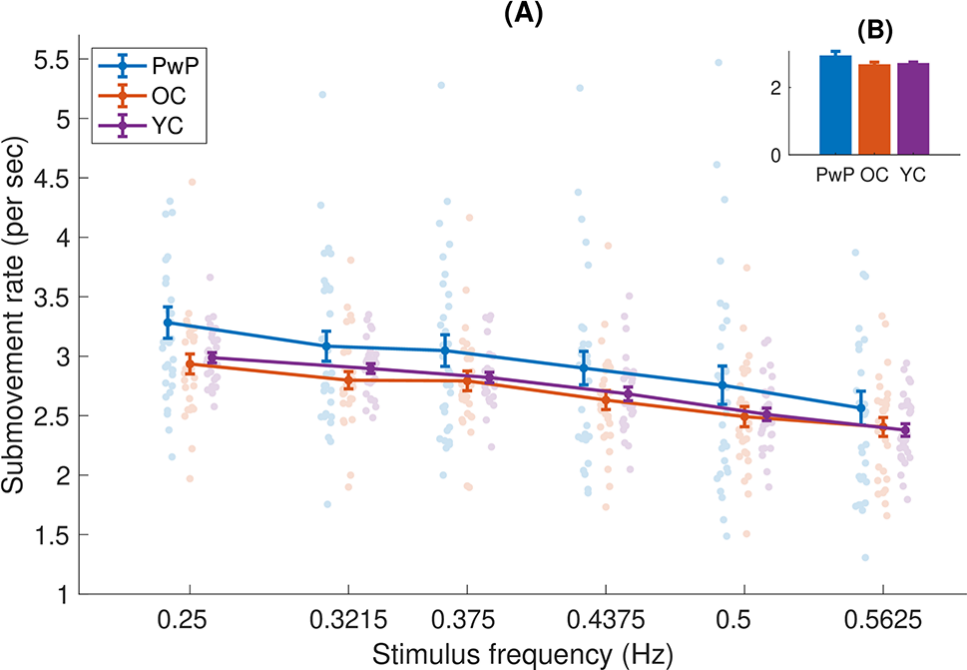
(**A**) Submovement rate (types 2 and 3 pooled together), as a function of frequency. Each dot is an individual participant, the dots are randomly shifted horizontally within a given frequency for clarity. (**B**) The inset shows the main effect of group (averaged across frequencies) - means and standard errors, with significant differences shown by horizontal black lines

**Fig. 5:**
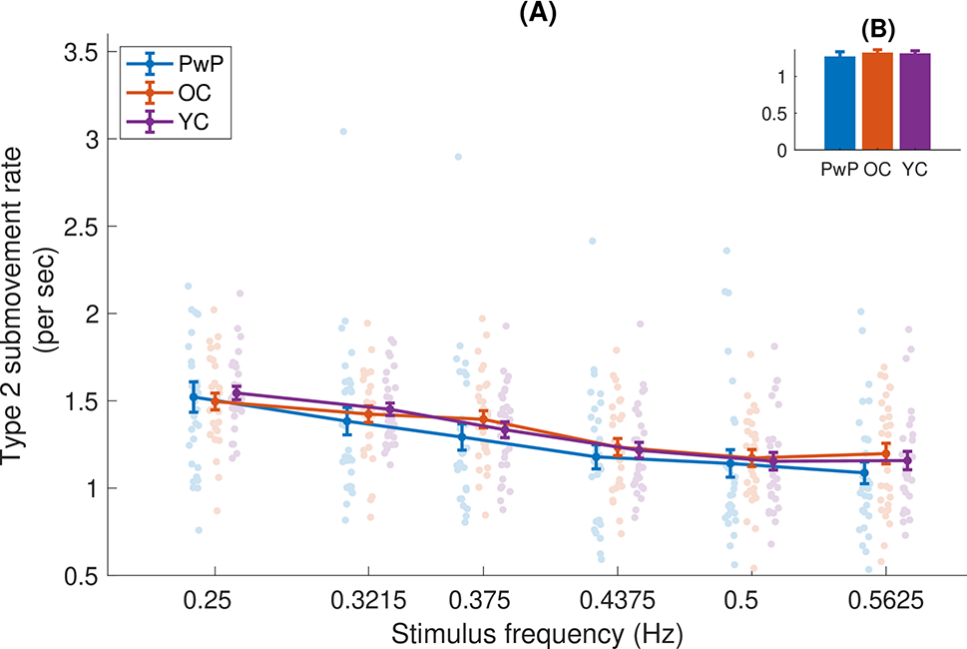
(**A**) Type 2 submovement (acceleration zero crossing) rate, as a function of frequency. Each dot is an individual participant, the dots are randomly shifted horizontally within a given frequency for clarity. (**B**) The inset shows the means and standard errors of the three groups, averaged across frequencies

One operational concern was the age of our PwP cohort. As in many other tasks [33], we expect to see a general reduction in tracking performance as people get older [34]. To control for the effect of age, we matched our PwP cohort with an age-matched older control cohort (OC). We also included a younger control (YC) cohort, in order to measure baseline performance on the task [35]. We expected to see better performance in YC compared to OC in all aforementioned measures [33].

To summarize our operational hypotheses, we expect to see:

1. An increase in submovement rates (type 2 and type 3) at lower frequencies, for all cohorts (PwP, OC, YC), as has been observed previously [6].
2. More submovements for PwP compared to age-matched participants (type 2, type 3: PwP > OC), based on previous studies [21].
3. As in most motor tasks, more submovements for older than younger controls (type 2, type 3: OC > YC), as is typically observed [36].
4. More spatial and timing tracking errors for PwP compared to age-matched controls (dX, dT: PwP > OC), due to a general increase in tracking errors in PwP [28].
5. Similar to 3, more tracking errors for older controls than younger controls (dX, dT: OC > YC), as previously observed [37].

## Methods

### Participants

Ninety participants took part in this cross-sectional study, in three groups: PwP (*N* = 31, 14 females, age 67 ± 11 years, disease duration 7.6 ± 4.6 years, range 1–22 years); a control group of older adults (OC: *N* = 29, 16 females, age 62 ± 11 years), and a control group of younger adults (YC: *N* = 30, 13 females, age 25 ± 6 years). The sample size was selected in order to observe differences in type 3 submovments, based on [21], where for small continuous movements, they found an incidence of 66.0 ± 2.9% for the control group, and 69.5 ± 4.2% for the PwP group. In order to find a significant difference between the two groups with a power of 0.95 in a t-test, we would require 29 participants per group [38]. For the PwP group, the inclusion criteria were a diagnosis of idiopathic Parkinson’s disease (clinically established or probable) according to the MDS Clinical Diagnostic Criteria [39]; bradykinesia of the right upper limb (according to the MDS-UPDRS part 3 examination of finger taps, hand movements, and rapid alternating movements) with the sum of the 3 item scores of ≥ 3. The participants in the PwP group could be either treated or untreated, including L-dopa, as long as they did not experience motor fluctuations or dyskinesia according to the relevant MDS-UPDRS items. Exclusion criteria for all groups were another neurological or orthopedic problem affecting the right upper limb, people with high competency in hand skills (e.g. musicians, dancers, sportspersons), and significant tremor or dyskinesia during drawing or writing. Before beginning the experiment, the procedure was explained, and all participants signed an informed consent form. The experiment received ethical approval from the Sheba Medical Center and the Tel Aviv University Institutional Review Boards. The experiment was performed at the Sheba Medical Center (PwP and OC groups) and Tel Aviv University (YC group), using the same equipment for all groups, between January 2020 and March 2021. All participants who started the experiment completed the protocol.

### Experimental procedure

The participants played the one-person mirror game [17]. The participants were instructed to track the movement of a sinusoidally oscillating red ellipse moving left and right. A sinusoidal movement was selected for the rhythmic movements used here, as for these movements, a sinusoid is the movement with the lowest mean squared jerk [40], as compared to the 5th order polynomial which has the lowest mean squared jerk for point to point movements [2]. They did so by moving a stylus on a graphics tablet that recorded their motion and controlled the location of a blue ellipse. Both ellipses were the same size (width 1.7 cm, height 3.2 cm) - the ellipse size, along with the gap between them, was selected to require only modest accuracy. The movements were constrained to be within a narrow channel so that only left-right movements could be made (see Fig. 2). The stimuli were presented and recorded on a Wacom Cintiq 27QHD combined graphics tablet and display, sampling the motion at 100 Hz, using Repeated Measures software [41]. The participants sat comfortably next to the tablet which was placed close to the edge of the table, with their legs under the table, but we did not control the trunk posture.

The stimuli were similar but not identical to those we used in previous experiments [6]. In particular, lower frequencies of movement (0.25–0.5625 Hz) were used to make the game more accessible to the older population. The kinematic parameters of the presented stimuli (i.e., their frequency and peak velocity, which defines the amplitude) can be found in Supplementary Table 2. Each participant performed 11 one-minute trials, and the frequency / peak velocity changed once or twice in each trial. The amplitudes (end-to-end) of the movements ranged from 6.3 cm to 33.2 cm. The red ellipse always started in the center of the screen, and did not move for 5 s to allow the participant to place the stylus in the appropriate location. Apart from the visual feedback of the two ellipses, no additional feedback on their performance was provided.

### Outcome measures

We calculated the rate of type 2 and type 3 submovements [14, 21, 42] as well as the total rate of submovements [21] (i.e., per second). For completeness, in the supplementary material, we also present the number of submovements per stroke. We did not calculate type 1 submovements because they are primarily involved in correcting for overshooting, which is unlikely to occur in a tracking task. We first filtered the left-right data with a two-way lowpass 4th order Butterworth filter with a cutoff of 4 Hz. We chose this relatively low cutoff frequency to avoid measuring tremor as submovements, to allow a fair comparison between participants with some tremor (primarily in the PwP group) and the other participants. For completeness, an analysis using a higher cutoff of 10 Hz, that is, a less strict filter, can be found in the supplementary material. We then calculated the velocity magnitude from the differences in stylus positions between subsequent samples from the left-right data. Type 2 submovements (peaks in the velocity profile, see Fig. 3) were identified from acceleration zero crossings from positive to negative accelerations. We did not include velocity magnitude peaks that were within 50 ms from a velocity magnitude peak in the stimuli. Type 3 submovements (re-acceleration - troughs in the acceleration profile when the acceleration is positive; or peaks in the acceleration profile when the acceleration is negative, see Fig. 3) were identified from jerk zero crossings from negative to positive jerk values when the acceleration was positive, or from positive to negative jerk values when the acceleration was negative.

In addition, we calculated two error measures, using the same techniques as in previous studies [6, 17]. For calculating these measures, the raw data was filtered with a two-way 4th order lowpass Butterworth filter with a cutoff of 10 Hz. Specifically, we first performed registration of the data to the stimulus [43]. The temporal difference between the stimulus and the data calculated in the registration procedure (i.e., the lag) is the mean timing error (dT). We then used the registered data to calculate the relative position error (dX): $\:dX=\frac{2}{n}{\sum\:}_{i=1}^{n}\frac{\left|{x}_{1}^{i}-{x}_{2}^{i}\right|}{\left|{x}_{1}^{i}+{x}_{2}^{i}-2{x}_{c}\right|}$ where $\:{x}_{1}^{i}$ and $\:{x}_{2}^{i}$ are the positions of the ellipse of the participant and the stimuli respectively at time i, after registration has taken place. For the denominator of the position error (dX), we subtract the location of the center x_c_, which is the midpoint of the sine waves, in order to normalize by the movement amplitude. When the denominator was small (< 10 pixels for dX), these values were not included in the sum to prevent instability in the measure. Note that due to their definitions, dT and dX are always zero or positive.

### Statistical analyses

We used JASP (Version 0.18.3) for the statistical analyses. The number of males and females in the older adult groups were compared using a chi-squared test, as were the younger and older adult groups, and the ages of the older adult groups were compared with a t-test. Mixed-design ANOVAs were used to compare submovement incidence and the dX and dT error measures, with a within-group factor of stimulus frequency (6 levels), and a between-group factor of group. We performed a similar analysis as a function of peak velocity - these results are presented in the Supplementary material. Significant differences between groups were analyzed with post-hoc t-tests, while significant effects of stimulus frequency were analyzed by comparing the slopes (linear regression lines of the relevant variable as a function of stimulus frequency) to 0 using t-tests. Holm correction was performed for post-hoc tests, the reported results are after the correction. When the assumption of sphericity was violated according to Mauchley’s test of sphericity, we used the Greenhouse-Geisser correction. While some of the data showed some small differences from a normal distribution and did not meet the assumption of homoscedasticity, we still used an ANOVA, as an ANOVA is relatively robust to deviations from normality [44] and homoscedasticity when the group sizes are approximately equal and greater than 30 participants [45, 46], as is the case here. We also ran a Bayesian mixed-design ANOVA to compare whether there is a difference in the number of submovements between older and younger participants (not including PwP), with the same factors as before. Results were considered statistically significant if *p* < 0.05, and a trend if 0.05 < *p* < 0.1. For the Bayesian ANOVA, we presented the Bayes factors. Unless otherwise specified, values presented are means ± standard errors. Cohen’s d was provided for effect size when relevant.

### Software and data availability

The software for performing the experiments is available online [41]. The datasets generated and analyzed during the current study are available in the figshare repository (10.6084/m9.figshare.22786244) [47].

## Results

### Participants

The PwP group had a score of 7.6 ± 3.8, range 2–14 (maximum possible 28), on the hand-related subset of the MDS-UPDRS [39], where higher scores correspond to more severe symptoms. Further details about the PwP group can be found in Supplementary Table 1. The number of males and females in the PwP and the older adults group was not found to differ significantly (χ^2^(1) = 0.27, *p* = 0.61), a similar finding was found for their ages (t(58) = 1.71, *p* = 0.09, d = 0.443), although a trend was observed as the mean age of the PwP (67.1 ± 1.9) was greater than that of the OC group (62.2 ± 2.1). We did not find a difference in the number of males and females between the OC and the YC groups (χ^2^(1) = 0.42, *p* = 0.52).

### People with Parkinson’s disease produce more type 3 submovements than older or younger controls in continuous tracking

As a first analysis, we looked at the rate of type 2 and type 3 submovements (pooled together) across different frequencies, for the 3 experimental groups (see Fig. 4). No main effect was observed for group (F(2,87) = 2.372, *p* = 0.099), although a trend was observed. Post-hoc tests did not show significant differences between the groups (all p > = 0.146). As expected, a main effect was observed for stimulus frequency (F(3.967,345.101) = 121.964, *p* < 0.001). As stimulus frequency increases, participants made less submovements. We confirmed this observation by calculating the slopes of the regression lines for each participant individually. We found using a t-test that the slopes were significantly less than 0 (t(89)=-17.86, *p* < 0.001, d=-1.87). There was no significant interaction of group and frequency (F(7.933,345.101) = 0.916, *p* = 0.502). A similar analysis was performed for the number of submovements per stroke (rather than the number of submovements per second), this analysis can be found in Supplementary materials.

We also examined the rate of type 2 and type 3 submovements separately. For type 2 submovements, we did not observe a significant difference across groups (F(2,87) = 0.272, *p* = 0.763). Again, a significant effect of stimulus frequency was observed (F(3.510,305.409) = 91.604, *p* < 0.001), such that there was a reduction of type 2 submovements with increasing stimulus frequency (see Fig. 5). Looking at the slopes of regression lines fit for each participant, we found that they were significantly less than 0 (t(89)=-14.29, *p* < 0.001, d=-1.49). Again, no interaction of group and frequency was observed (F(7.021,305.409) = 1.017, *p* = 0.419).

**Fig. 6:**
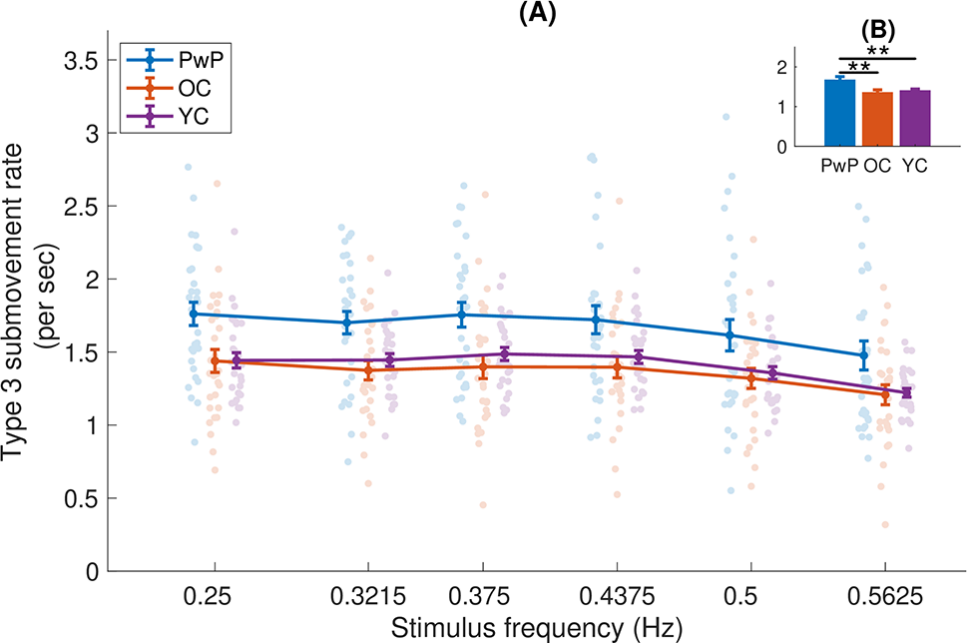
(**A**) Type 3 submovement (jerk zero crossing) rate, as a function of frequency. Each dot is an individual participant, the dots are randomly shifted horizontally within a given frequency for clarity. (**B**) The inset shows the main effect of group (averaged across frequencies) - means and standard errors, with significant differences shown by horizontal black lines. ** indicates significantdifferences at the level of *p* < 0.01

In contrast, type 3 submovements did show a main effect of group (F(2,87) = 6.751, *p* = 0.002, see Fig. 6). Post-hoc tests showed that PwP produced significantly more type 3 submovements (1.67 ± 0.06 submovements/sec) than both the OC group (1.36 ± 0.07 submovements/sec, t(87) = 3.383, *p* = 0.003, d = 0.80), and the YC group (1.40 ± 0.07 submovements/sec, t(87) = 2.902, *p* = 0.009, d = 0.68). No difference was observed between OC and YC groups (t(87)=-0.502, *p* = 0.617, d=-0.12). A significant effect of stimulus frequency was again observed (F(3.219,280.051) = 25.949, *p* < 0.001). The slopes of regression lines fit to each participant were significantly less than 0 (t(89)=-6.05, *p* < 0.001, d=-0.64). There was no interaction between frequency and group (F(6.438,280.051) = 0.419, *p* = 0.878).

### People with Parkinson’s have higher error rates than controls

**Fig. 7:**
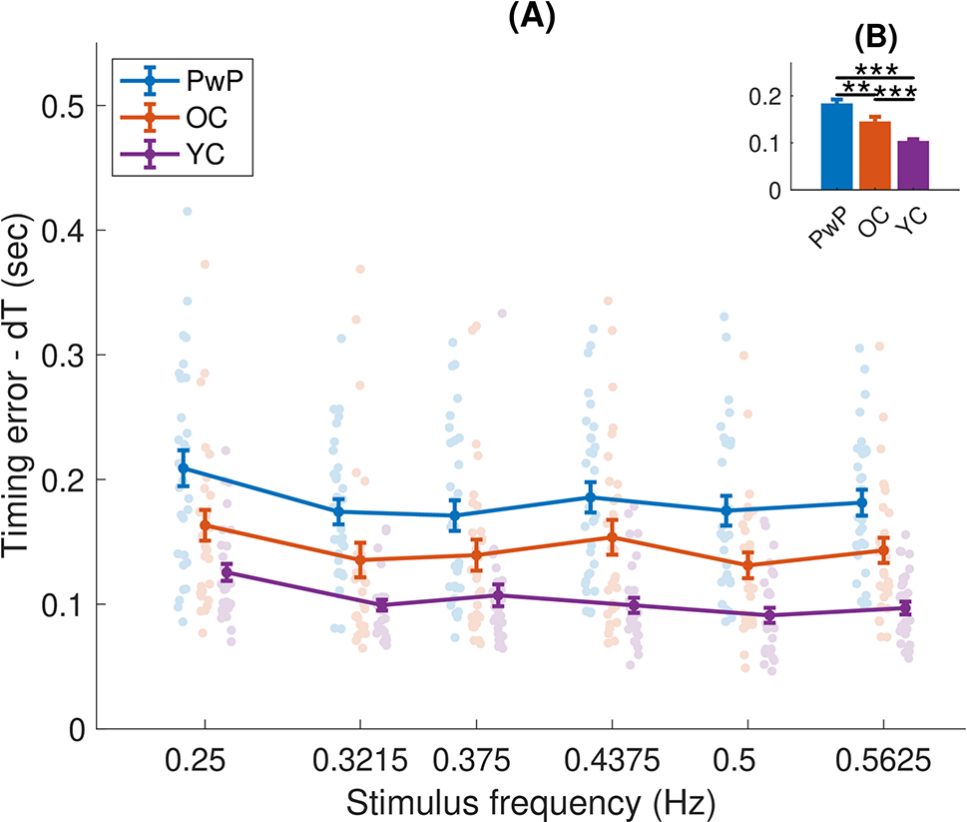
(**A**) Mean timing error (dT), as a function of frequency. Each dot is an individual participant, the dots are randomly shifted horizontally within a given frequency for clarity. (**B**) The inset shows the main effect of group (averaged across frequencies) - means and standard errors, with significant differences shown by horizontal black lines. ** indicates significant differences at the level of *p* < 0.01, *** at the level of *p* < 0.001

We observed clear differences between the groups in terms of the error measures, dT and dX. In terms of the mean timing error dT (Fig. 7), differences between the groups are shown by a main effect of group in the mixed-design ANOVA (F(2,87) = 13.054, *p* < 0.001). Specifically, PwP (0.183 ± 0.009) had significantly greater timing errors (dT) than the OC group (0.147 ± 0.009; t(58) = 2.874, *p* = 0.005, d = 0.63), who in turn had significantly greater timing errors than the YC group (0.102 ± 0.009; t(57) = 0.787, *p* = 0.001, d = 0.79). A main effect of frequency was also observed (F(4.206,365.928) = 10.694, *p* < 0.001). Post-hoc tests showed that dT for the lowest frequency (0.25 Hz) was higher than that for all other frequencies (all *p* < 0.001); the other frequencies did not show significant differences from each other (all *p* > 0.05). No interaction of group and frequency was observed (F(8.412,365.928) = 0.724, *p* = 0.677).

**Fig. 8:**
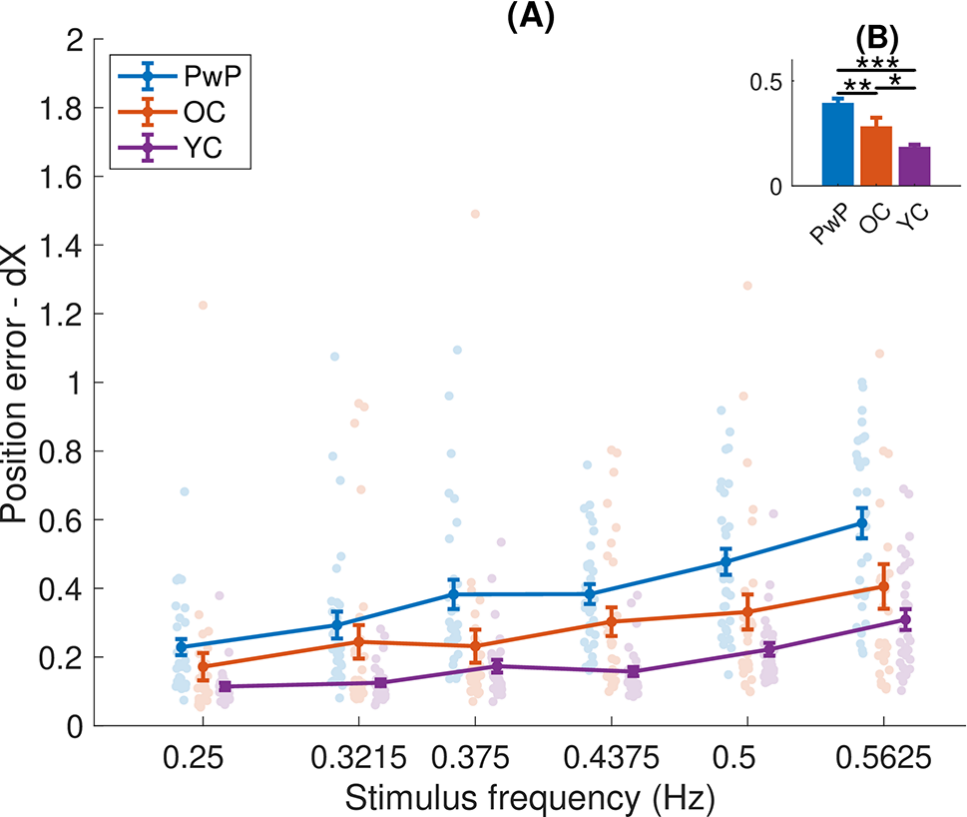
(**A**) Relative position error (dX), as a function of frequency, presented for the 3 groups. Each dot is an individual participant, the dots are randomly shifted horizontally within a given frequency for clarity. (**B**) The inset shows the main effect of group (averaged across frequencies) - means and standard errors, with significant differences shown by horizontal black lines. * indicates significant differences at the level of *p* < 0.05, ** at the level of *p* < 0.01, *** at the level of *p* < 0.001

For the relative position error (dX), the PwP group showed greater errors than the other two groups (Fig. 8), as shown by a main effect of group (F(2,87) = 13.364, *p* < 0.001). Post-hoc tests showed that the PwP group (0.389 ± 0.029) had larger position errors than the OC group (0.265 ± 0.029; t(58) = 2.995, *p* = 0.007, d = 0.63), which in turn had larger position errors than the YC group (0.181 ± 0.029; t(57) = 2.019, *p* = 0.047, d = 0.43). A main effect of frequency was also observed (F(3.839,333.959) = 40.882, *p* < 0.001), but in the opposite direction of the timing error - the relative position error became larger with higher frequencies. This was confirmed by testing whether the regression lines were positive (t(89) = 12.56, *p* < 0.001, d = 1.31). An interaction of group and frequency was also observed (F(7.677,333.959) = 2.116, *p* = 0.036). We compared regression lines fitted to each participant and found that the PwP group had a steeper slope (1.16 ± 0.73 per Hz) compared to both the OC (0.75 ± 0.49 per Hz; t(58) = 2.56, *p* = 0.026, d = 0.65) and YC (0.53 ± 0.43 per Hz; t(59) = 4.06, *p* < 0.001, d = 1.02) groups. The difference between OC and YC was not significant (t(57) = 1.77, *p* = 0.081, d = 0.456), although a trend was observed.

### Older adults produce the same rate of submovements as younger adults across a range of frequencies

To verify the lack of difference between OC and YC groups in terms of the number of submovements, we performed an additional Bayesian analysis with only the younger (YC) and older (OC) control groups, in order to quantify how much more likely the hypothesis that only frequency affects the submovement measures (i.e., no difference between groups) is compared to the alternative hypothesis (that there is a significant difference between groups and/or an interaction), see Supplementary Tables 3 to 5 for the full results of the analyses.

For the three measures presented here (total number of submovements, number of type 2 submovements, and number of type 3 submovements), the model only dependent on frequency best explained the data - better than a model including group (2.34 times more likely for the combined measure, 2.65 times more likely for type 2 submovements, and 2.06 times more likely for type 3 submovements). The full tables are provided in the supplementary material.

## Discussion

### Summary of main findings

In this study, we measured the motor performance in a manual tracking task of PwP, and contrasted it with the behavior of age-matched older controls (OC) and younger controls (YC). Our main focus was the rate of submovements across a range of tracked stimuli, in particular for low frequencies, where previous works reported a breakdown of smoothness. We employed two measures of submovement rate previously used in the literature (type 2 and type 3, see methods). We also measured two types of performance errors: mean timing error (dT) and relative position error (dX).

Confirming the results of previous studies, we observed a consistent decrease in submovement rates as the frequency of the tracked stimulus increased (Fig. 4), in accordance with our first hypothesis. Comparing the three cohorts, we see that while significant differences were not observed for the overall submovement rate, the PwP group produced more submovements than the other two groups for type three submovements, partially confirming our second hypothesis. Interestingly, this is not the case for type 2 submovements (compare Figs. 5 and 6), highlighting the importance of measuring different types of submovements. We note that these differences hold despite controlling here for movement duration [27], indicating that the increased number of submovements in PwP is not solely due to longer movement times. These effects occurred across the frequency spectrum tested in the current study and were not specific to low movement frequencies. In addition, we did not see any differences in the rate of submovements between OC and YC, contrary to our third hypothesis.

Focusing on error measures, we observed the expected trends where the PwP cohort made more mean timing errors (Fig. 7) and relative position errors (Fig. 8) than OC, who in turn made more mean timing and relative position errors than YC, confirming our fourth and fifth hypotheses. For all groups, we also observed a decrease in mean timing errors and an increase in relative position errors, as stimulus frequency increased. One main difference was observed between the two error measures - an interaction of group and frequency was observed only for dX but not dT - the increase in relative position error (with increasing frequency) showed different slopes across groups, whereas the slopes for dT were similar ( i.e. relative position error is not just overall worse in older adults and PwP, but the differences in error become greater at higher frequencies).

### Insights into submovements

Going back to the question we posed in the introduction, it seems that two things are clear: (1) the consistent relationship between stimulus frequency and rate of submovements that was observed in several studies before [5, 6, 17] holds also for PwP; (2) PwP perform more submovements than both older and younger control participants, in a constant way across different frequencies. The reduction of smoothness (quantified by an increase in the number of submovements) for PwP was similar for slow, medium and fast movements (no interaction in Fig. 5). In other words, we did not find any evidence that PwP are particularly better (or worse) in producing slow and smooth strokes. The increased use of slower movements in PwP, which may be due to increased difficulty in switching between static and dynamic states [48], does not lead to an improvement in how these movements are performed. Our results do not allow us to conclude whether the lack of improvement in performing slow movements is a result of the disease, or is a more general limitation in general for producing slow, smooth movements [7].

Interestingly, the significant difference between groups was only observed for type 3 and not type 2 submovements, similar to the results of Dounskaia et al. [21]. Type 3 submovements may be considered as the most subtle of the submovements - whereas type 2 submovements represent peaks and troughs in the velocity profile, type 3 submovements represent inflection points in the velocity profile. It may be due to the increased error rate in PwP, more of these subtle corrections are made, which are less necessary for the other groups.

### Why do PwP produce more submovements across different frequencies?

Previous studies have shown that PwP made more submovements in point-to-point reaching tasks [19, 21–23]. We speculate that PwP need to more deliberately plan their movements, resulting in more submovements, as a result of reduced automaticity. PwP need greater neural activity in movement-related areas compared to controls when performing automatic movements, with this hyperactivation possibly being compensation for basal ganglia dysfunction [49]. As we learn new movements, they become more automatic [50], and with learning, we observe a reduction in the number and complexity of submovements [51, 52], a process that appears to be compromised in PwP. Automaticity is typically tested by performing a dual-task (e.g., simultaneously counting backward by sevens). If performance on the motor task is not impaired, the motor task is considered to be automatic [49]. Previous studies, using other tracking tasks, have shown greater dual-task costs for PwP compared to age-matched controls [53, 54]. The mechanism suggested in this work can be further tested by combining our tracking task with measures of cognitive load [e.g. 55].

It may also be that PwP used some sort of cue from the observed movement, such as the extremes of the movement of the stimulus (an ellipse-shaped cursor), since external cues can improve movement production in PwP [56]. In a task requiring moving between multiple targets, PwP were significantly worse when cues were removed compared to a control group, with errors accumulating in the sequence [57]. Occluding feedback at times of particularly salient information might be a way to further test this possibility in our tracking task.

The observation that PwP generated more submovements in continuous tracking suggests that they are not automatically correcting their trajectories, as occurs in double-step experiments with small jumps, where people are not consciously aware of the corrections they make [58]. The conscious control of submovements would likely lead to a longer delay (compared to automatic corrections), thus necessitating more submovements due to increased errors. We note that this tracking task differs from the types of tasks previously studied, which involved mainly reaching or point-to-point movements. In a tracking task, continuous visual feedback on performance is available (i.e., one can continuously see if they are aligned with the tracked stimulus or not), requiring only a short planning horizon. In contrast, in point-to-point tasks, the participant is generally required to plan a movement all the way to the target and is not given guidance on how to execute the movement. Additionally, these two types of tasks (continuous vs. discrete) likely involve different planning processes [59]. For example, in point-to-point reaching tasks, the participant needs to stop at the target, and some of the additional submovements observed in these tasks may be due to a deficit in smooth movement termination observed in PwP [21], whereas in a continuous tracking task such as the one used in this experiment, participants do not need to stop (but rather, reverse movement direction).

The differences in submovement rate, and in particular, the increase in type 3 submovements, may be a tool for differentiating between healthy aging and early signs of Parkinson’s disease, in the prodromal phase. This possibility would require further examination and retrospective analyses of movement data from people later diagnosed with Parkinson’s disease.

A potential alternative explanation for the differences in submovement rate is that PwP have a modified error deadzone [16, 60]. An error deadzone is a region surrounding the target where corrections are not made, i.e., small errors are ignored. In PwP, an overreliance on visual feedback [61], which may be related to poorer proprioception [62] or impaired multisensory integration [63] could lead to more corrections as they may not avoid producing unnecessary corrective movements due to difficulty in identifying when the movement is “close enough” to the desired location, i.e., within the deadzone.

It may rather be that the increased number of submovements observed in PwP stems from the increased errors that they make, and a desire to perform the task as well as they can. This possibility could be tested by altering the nature of the feedback provided so that the participants are less able to judge their error, such as moving the stimulus and target further apart, by performing an “anti-tracking” task where the participant move the ellipse in the opposite direction to the stimuli, or by occluding feedback during parts of the movement.

### The range of studied slow and smooth strokes

Previous studies have shown that people display significant deviations from typical arm movement patterns when they move slowly. In some of these studies, the instructions were to move slowly [64, 65], to move at a constant slow speed [8], or to move slowly and smoothly [66, 67]. Similarly, the current study showed a decline in the number of submovements as a function of frequency, that is, greater smoothness at higher frequencies. It is interesting to note that the slopes for PwP are very similar to OC and YC - no interaction of group and frequency was observed. The reduction in number of submovements in this study is less dramatic than in previous studies (see Fig. 1) because of the reduced range of frequencies used here (0.25 Hz to 0.565 Hz) compared to previous studies where we used frequencies from 0.25 to 1.0 Hz [6, 68]. We chose this range because it is harder for older adults and PwP to perform these faster movements.

### Complementary effects of frequency on dT and dX

We observed across participants that the mean timing error (dT) and the relative position error (dX) showed complementary effects as a result of frequency, where dT decreased at higher frequencies, whereas dX increased at higher frequencies. The decrease in dT as the frequency increased is likely due to an improved ability to time shorter intervals [69]. The increase in dX with increasing frequency is likely a result of the speed-accuracy tradeoff. As higher frequency movements have shorter durations, the speed-accuracy tradeoff predicts lower accuracy (i.e., higher dX) [70]. Interestingly, an interaction was observed here - PwP were even more affected during relatively faster movements. This may be due to an avoidance of fast movements observed in PwP, likely due to a shift in the cost/benefit ratio for making fast movements [71]. In this study, PwP were more likely to select slower movements despite being capable of making movements in a similar speed range as age-matched controls. It is not clear from these experiments whether these differences are due to differences in perception of speed [63] or in selection of execution velocity.

### Older adults produce more tracking errors than younger adults

As expected, our results show more tracking errors for older adults than younger adults (see Figs. 7 and 8). This finding is in agreement with previous studies showing that older adults make more tracking errors [34], likely due to deficits in the medial-frontal error evaluation system. This may reflect a deficit in identifying, assessing, and reacting to motor errors observed in older adults [72]. In a sinusoidal tracking task, older participants lagged more than younger adults and showed overall lower velocity [73], corresponding to the larger errors observed in this study. While PwP are able to perform tracking tasks and make the short-term predictions necessary to succeed in this task [29], larger errors are typically observed in tracking tasks in PwP compared to age-matched controls [74]. Additionally, PwP tend to use slower velocity when performing the task [30]. These larger errors were observed here - we found larger errors (compared to older adults) in both the mean timing error (dT) and the relative position error (dX).

### Lack of difference in submovement rate between younger and older control groups

A somewhat surprising finding is that the rate of submovements was not found to be different between the OC and YC groups (see Fig. 4). This is surprising because differences in performance are usually observed in many motor tasks between younger and older adults [33], as was indeed observed here in timing and spatial errors (see Figs. 7 and 8). A novel observation made in this study was a lack of difference in the submovement rate between the younger and older adults in the control groups (see Fig. 4). This may not have been previously observed, because when studying other types of movement (such as point-to-point movements), older adults generally make slower movements, i.e., with lower vigor [75], which lead to longer duration movements and hence more submovements. The results of our study suggest that OC prefer to make less submovements at the cost of higher error rates: it is likely they could increase their submovement rate (as PwP do) but they choose not to do so. It may be that making more submovements may be more costly as they result in less smooth movements [1], greater physical effort [75], and an increase in cognitive load [76]. It is possible that PwP are less able to take into account these factors, causing them to produce more submovements.

This lack of change in submovement rate over age that occurred despite the decline observed in many other measures (including the error measures in this study) suggests that the rate of submovement production is relatively stable across ages [see 17]. The dissociation observed here between submovement rate and the error measures (dX and dT) provides further support for the notion that submovements are not solely a result of error correction mechanisms [14, 21, 36].

For type 3 submovements, a significant difference was observed between PwP and both OC and YC groups. However, from the graph (Fig. 6), it appears that YC make slightly more type 3 submovements than OC, although the difference is small and not significant. This may be due to a “conversion” of type 2 submovements to type 3 submovements, as we observed in a previous study [77]. Type 2 submovements correspond to peaks and troughs in the velocity profile, whereas type 3 submovements correspond to inflection points. It may be that due to the better ability of the YC to control their movements, while they are unable to not produce submovements at all, they can produce a greater proportion of type 3 submovements, which would result in smoother movements and may explain the increased rate of type 3 submovements in this group.

### Further directions, limitations and conclusions

In general, understanding the limits of slow and smooth strokes is an interesting question, because there are tasks that require these movements. For example, when playing string instruments, the musician is often required to make very slow bowing movements that need to be smooth in order to produce the required sound. Another example was presented in a paper from Doeringer & Hogan [8]: when using an unpowered lathe, two-handed techniques are necessary to achieve smooth motion of the machine [78]. Understanding how experts move slowly in these practices, despite the seemingly universal human challenge with slow and smooth strokes, is an intriguing avenue for further research.

In addition, follow-up studies can try to unravel the underlying mechanisms leading to the fairly constant smoothness/frequency slope observed in the current work and previous studies. We recently showed that this phenomenon can not be explained solely by lack of previous experience with slow and smooth strokes, as both people who performed an intensive motor learning protocol, and experts in slow motions (Tai Chi experts), also showed this characteristic slope [77]. Another possible follow-up can test the hypothesis that the smoothness-frequency relationship is determined (to some degree) by the physical properties of the moving body part. This can be done, for example, by asking people to track the same stimuli with body parts that have different mass and inertia, such as the finger, arm, leg and whole trunk.

There are several limitations to the current work. First, the PwP group’s disease heterogeneity limited the drawing of more general conclusions about the performance of PwP. A second limitation is the relatively low MDS-UPDRS scores of the participants. A possible follow-up will include a larger sample of PwP, with a wider range of MDS-UPDRS scores. Third, as different PwP participants had differences in L-dopa dose, there may have been differences in subsequent mood which may have affected performance. Fourth, the younger and older control groups were recruited from different populations: younger controls were mostly university students, compared to the older controls who came from a wider range of backgrounds.

In this study, we found that PwP produced larger errors compared to both younger and older control groups, in both timing and position, when performing a tracking task. In addition, they produced more submovements. Importantly, we note that this difference in submovement rate is not due to increased movement duration, which was controlled due to the nature of the activity. The findings of this study can help inform the selection of the frequency of arm movement that are most appropriate for PwP to perform to maximize smoothness (i.e., minimize submovement rate) while simultaneously minimizing error.

## Electronic supplementary material

Below is the link to the electronic supplementary material.

Supplementary Material 1

## Data Availability

The software for performing the experiments is available online (https://doi.org/10.5281/zenodo.10438). The datasets generated and analyzed during the current study are available in the figshare repository (10.6084/m9.figshare.22786244).

## Abbreviations

dX: Relative position error
dT: Mean timing error
PwP: People with Parkinson’s disease
