# Supplementary material for: Submovements in manual tracking: people with Parkinson’s disease produce more submovements than age-matched controls
Source: J Neuroeng Rehabil. 2025 Mar 6;22:51. doi: 10.1186/s12984-025-01592-1 (PMC11884197; doi:10.1186/s12984-025-01592-1)
Supplement: Supplementary file 1 — Supplementary Material 1 [file 12984_2025_1592_MOESM1_ESM.docx]

**Supplementary data**

Supplementary table 1. Details of the People with Parkinson’s disease (PwP) group

| Participant | Age | Sex | LEDD | Years since first motor symptoms | Rest tremor amplitude | Postural tremor amplitude | Kinetic tremor amplitude | Hand rigidity | Finger tapping | Hand movements | Rapid alternating movements |
| --- | --- | --- | --- | --- | --- | --- | --- | --- | --- | --- | --- |
| PwP1 | 75.1 | F | 0 | 2 | 0 | 0 | 0 | 0 | 1 | 1 | 1 |
| PwP2 | 72.9 | F | 100 | 3 | 0 | 0 | 0 | 0 | 1 | 1 | 1 |
| PwP3 | 70.1 | M | 150 | 4 | 1 | 1 | 1 | 2 | 0 | 0 | 0 |
| PwP4 | 72.7 | M | 550 | 16 | 0 | 1 | 1 | 2 | 1 | 1 | 1 |
| PwP5 | 68.3 | M | 631.75 | 12 | 0 | 1 | 0 | 0 | 2 | 2 | 2 |
| PwP6 | 72.7 | F | 375 | 10 | 0 | 0 | 0 | 1 | 2 | 1 | 2 |
| PwP7 | 72.9 | F | 350 | 5 | 0 | 1 | 1 | 0 | 2 | 2 | 2 |
| PwP8 | 70.0 | F | 650 | 7 | 0 | 0 | 0 | 0 | 3 | 3 | 3 |
| PwP9 | 74.1 | F | 350 | 6 | 1 | 1 | 1 | 1 | 3 | 3 | 3 |
| PwP10 | 55.1 | M | 475 | 5 | 1 | 1 | 1 | 2 | 2 | 2 | 2 |
| PwP11 | 72.9 | M | 775 | 5 | 0 | 1 | 1 | 1 | 3 | 2 | 2 |
| PwP12 | 73.6 | F | 600 | 7 | 0 | 1 | 1 | 0 | 3 | 3 | 2 |
| PwP13 | 77.1 | F | 180 | 10 | 1 | 0 | 0 | 3 | 3 | 3 | 3 |
| PwP14 | 62.5 | M | 420 | 5 | 0 | 0 | 1 | 1 | 2 | 2 | 2 |
| PwP15 | 68.1 | F | 400 | 8 | 0 | 0 | 0 | 0 | 2 | 2 | 2 |
| PwP16 | 57.1 | M | 100 | 7 | 0 | 0 | 0 | 0 | 1 | 1 | 1 |
| PwP17 | 77.2 | M | 300 | 8 | 0 | 0 | 0 | 0 | 2 | 1 | 1 |
| PwP18 | 74.1 | M | 1304 | 13 | 0 | 0 | 0 | 1 | 1 | 1 | 1 |
| PwP19 | 70.2 | M | 150 | 1 | 1 | 1 | 1 | 0 | 2 | 2 | 1 |
| PwP20 | 68.3 | M | 460 | 5 | 1 | 0 | 1 | 2 | 2 | 2 | 2 |
| PwP21 | 33.7 | M | 100 | 3 | 1 | 1 | 1 | 2 | 3 | 3 | 3 |
| PwP22 | 66.8 | M | 300 | 4 | 0 | 0 | 0 | 1 | 1 | 1 | 1 |
| PwP23 | 78.5 | M | 1135.5 | 22 | 0 | 1 | 1 | 1 | 2 | 3 | 3 |
| PwP24 | 86.0 | F | 440 | 14 | 0 | 0 | 0 | 0 | 0 | 1 | 1 |
| PwP25 | 57.9 | F | 337 | 5 | 0 | 0 | 0 | 1 | 1 | 1 | 1 |
| PwP26 | 56.5 | M | 1930 | 15 | 0 | 0 | 0 | 0 | 1 | 1 | 1 |
| PwP27 | 58.2 | F | 100 | 4 | 0 | 0 | 0 | 0 | 1 | 1 | 1 |
| PwP28 | 70.1 | M | 0 | 5 | 1 | 1 | 1 | 2 | 3 | 3 | 2 |
| PwP29 | 59.4 | M | 2452 | 10 | 0 | 1 | 1 | 2 | 2 | 2 | 2 |
| PwP30 | 79.2 | M | 898 | 8 | 0 | 1 | 0 | 1 | 4 | 3 | 3 |
| PwP31 | 49.1 | F | 0 | 7 | 1 | 2 | 1 | 1 | 3 | 2 | 2 |

*All motor tests are for the dominant (right) hand. LEDD is the Levodopa equivalent daily dose. The last 7 columns refer to parts of the MDS-UPDRS (scores range from 0=least affected to 4=most affected)*

Supplementary table 2. Stimulus properties: frequency and peak velocity. First-, second- and final-third correspond to the periods of approximately 0 - 20 seconds, 20 - 40 seconds, and 40 - 60 seconds, respectively. Frequency refers to the time taken for a full period of a sine wave. The amplitude of the movement can be calculated by $A=\frac{v_{peak}}{\pi f}$ *, where v_peak_ is the peak velocity, f is the frequency.*

| Stimulus number | First-third | Second-third | Final-third |
| --- | --- | --- | --- |
| 1 | 0.25 Hz, 22.53 cm/s | | 0.375 Hz, 15.02 cm/s |
| 2 | 0.375 Hz, 11.26 cm/s | 0.25 Hz, 22.53 cm/s | 0.5625 Hz, 15.02 cm/s |
| 3 | 0.5 Hz, 26.28 cm/s | 0.25 Hz, 18.77 cm/s | 0.25 Hz, 26.28 cm/s |
| 4 | 0.5 Hz, 15.02 cm/s | 0.5625 Hz, 22.53 cm/s | 0.5 Hz, 26.28 cm/s |
| 5 | 0.4375, 26.28 cm/s | 0.5 Hz, 15.02 cm/s | 0.3125 Hz, 22.53 cm/s |
| 6 | 0.3125 Hz, 11.26 cm/s | 0.3125 Hz, 11.26 cm/s | 0.4375, 22.53 cm/s |
| 7 | 0.3125 Hz, 18.77 cm/s | 0.5625 Hz, 11.26 cm/s | 0.3125 Hz, 26.28 cm/s |
| 8 | 0.5625 Hz, 26.28 cm/s | 0.5 Hz, 11.26 cm/s | 0.4375, 15.02 cm/s |
| 9 | 0.375 Hz, 18.77 cm/s | 0.4375, 18.77 cm/s | 0.5 Hz, 11.26 cm/s |
| 10 | 0.3125 Hz, 15.02 cm/s | 0.5625 Hz, 18.77 cm/s | 0.375 Hz, 26.28 cm/s |
| 11 | 0.5 Hz, 22.53 cm/s | 0.375 Hz, 18.77 cm/s | 0.4375, 11.26 cm/s |

Supplementary table 3. Bayesian Repeated Measures ANOVA results for combined type 2 and type 3 submovements.

| **Model Comparison** | | | | | | | | | | | |
| --- | --- | --- | --- | --- | --- | --- | --- | --- | --- | --- | --- |
| **Models** | | **P(M)** | | **P(M\|data)** | | **BF_M_** | | **BF_10_** | | **error %** | |
| Frequency |  | 0.200 |  | 0.690 |  | 8.887 |  | 1.000 |  |  |  |
| Frequency + group |  | 0.200 |  | 0.294 |  | 1.664 |  | 0.426 |  | 5.322 |  |
| Frequency + group + Frequency ✻  group |  | 0.200 |  | 0.017 |  | 0.067 |  | 0.024 |  | 11.639 |  |
| Null model (incl. subject and random slopes) |  | 0.200 |  | 7.211×  10^-56^ |  | 2.884×  10^-55^ |  | 1.046×  10^-55^ |  | 0.668 |  |
| group |  | 0.200 |  | 2.308×  10^-56^ |  | 9.233×  10^-56^ |  | 3.347×  10^-56^ |  | 0.875 |  |
|  | | | | | | | | | | | |
| *Note.*  All models include subject, and random slopes for all repeated measures factors. | | | | | | | | | | | |

Supplementary table 4. Bayesian Repeated Measures ANOVA results for type 2 submovements.

| **Model Comparison** | | | | | | | | | | | |
| --- | --- | --- | --- | --- | --- | --- | --- | --- | --- | --- | --- |
| **Models** | | **P(M)** | | **P(M\|data)** | | **BF_M_** | | **BF_10_** | | **error %** | |
| Frequency |  | 0.200 |  | 0.695 |  | 9.127 |  | 1.000 |  |  |  |
| Frequency + group |  | 0.200 |  | 0.262 |  | 1.422 |  | 0.377 |  | 4.868 |  |
| Frequency + group + Frequency ✻  group |  | 0.200 |  | 0.042 |  | 0.178 |  | 0.061 |  | 9.578 |  |
| Null model (incl. subject and random slopes) |  | 0.200 |  | 1.692  ×10^-46^ |  | 6.769  ×10^-46^ |  | 2.434  ×10^-46^ |  | 0.470 |  |
| group |  | 0.200 |  | 5.022  ×10^-47^ |  | 2.009  ×10^-46^ |  | 7.223  ×10^-47^ |  | 0.764 |  |
|  | | | | | | | | | | | |
| *Note.*  All models include subject, and random slopes for all repeated measures factors. | | | | | | | | | | | |

Supplementary table 5. Bayesian Repeated Measures ANOVA results for type 3 submovements.

| **Model Comparison** | | | | | | | | | | | |
| --- | --- | --- | --- | --- | --- | --- | --- | --- | --- | --- | --- |
| **Models** | | **P(M)** | | **P(M\|data)** | | **BF_M_** | | **BF_10_** | | **error %** | |
| Frequency |  | 0.200 |  | 0.665 |  | 7.948 |  | 1.000 |  |  |  |
| Frequency + group |  | 0.200 |  | 0.322 |  | 1.904 |  | 0.485 |  | 4.064 |  |
| Frequency + group + Frequency ✻  group |  | 0.200 |  | 0.012 |  | 0.050 |  | 0.018 |  | 8.848 |  |
| Null model (incl. subject and random slopes) |  | 0.200 |  | 1.426  ×10^-14^ |  | 5.703  ×10^-14^ |  | 2.143  ×10^-14^ |  | 0.436 |  |
| group |  | 0.200 |  | 6.120  ×10^-15^ |  | 2.448  ×10^-14^ |  | 9.200  ×10^-15^ |  | 2.766 |  |
|  | | | | | | | | | | | |
| *Note.*  All models include subject, and random slopes for all repeated measures factors. | | | | | | | | | | | |

**Supplementary results – Using a 10Hz lowpass filter**

There is a concern that some of the results observed may be due to using a relatively “heavy” filter, namely a two-way, 4^th^ order, 4 Hz lowpass Butterworth filter. We selected this filter with its relatively low cutoff frequency to remove the effects of tremor as this is not the focus of this study. Moreover, we are interested in submovement planning and execution rather than tremor, hence the appropriateness of the filter. Here, for completeness, we present the results of the submovement rates using a 10 Hz filter and compare them to the results for 4 Hz. The results for the error measures (dX and dT) already used a 10 Hz filter and so are not repeated here. Supplementary Figure 1 demonstrates the difference between the two filters – as the 10 Hz signal is ”noisier”, many more submovements are identified.

| *4 Hz* | *10 Hz* |
| --- | --- |
| *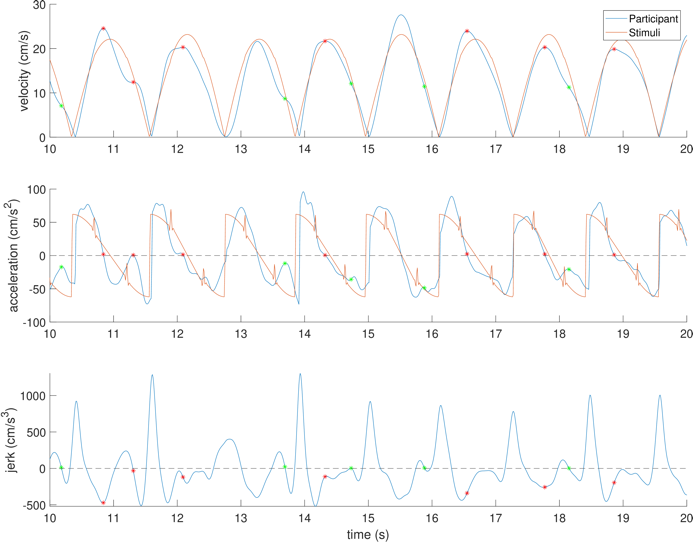* | 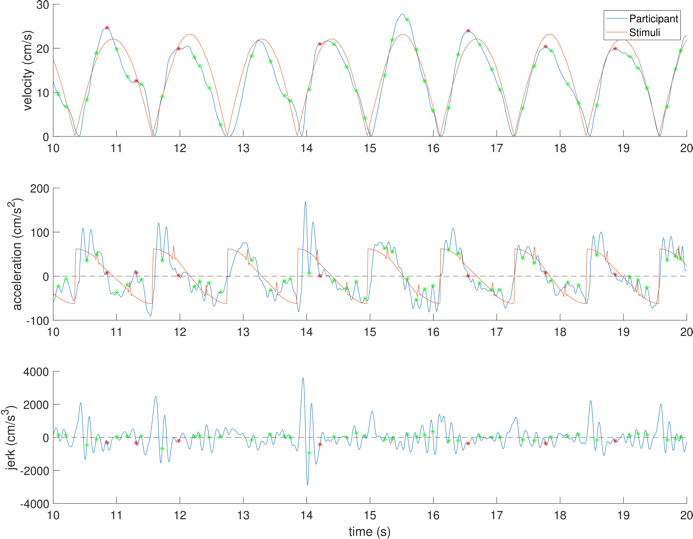 |

*Supplementary Figure 1. A typical example of the submovement calculations for the same participant and trial using a 4 Hz and 10 Hz lowpass Butterworth filter. The red stars indicate type 2 submovements, and the green stars indicate type 3 submovements.*

*Submovement rate (combined type 2+3)*

| *4 Hz* | *10 Hz* |
| --- | --- |
| *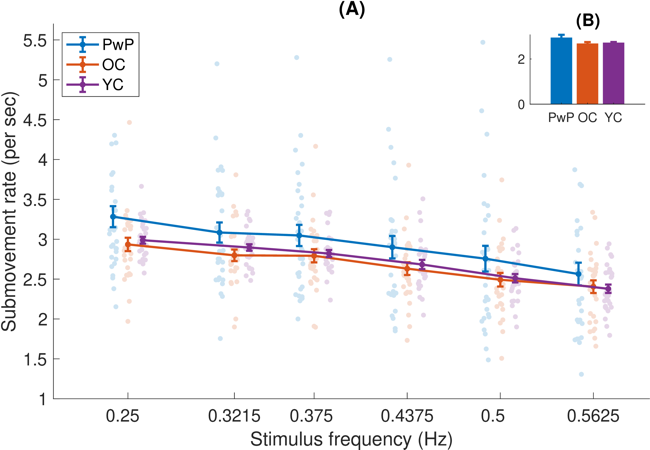* | 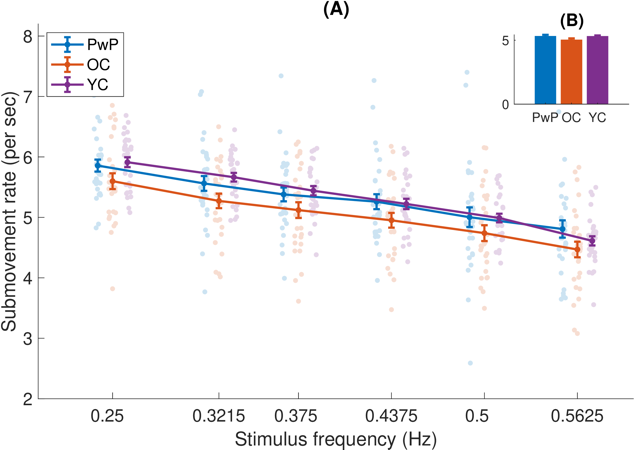 |

*Supplementary figure 2. Comparison of submovement rate (type 2 and 3 combined) with a 4 Hz and 10 Hz lowpass Butterworth filter. The inset shows the mean rate across frequencies.*

*Supplementary table 6. Results of a mixed-design ANOVA on the combined submovement rate, with both a 4 Hz and 10 Hz lowpass Butterworth filter.*

|  |  | 4 Hz | 10 Hz |
| --- | --- | --- | --- |
| Group | F | F(2,87) = 2.372 | F(2,87) = 2.418 |
|  | p = | 0.099 | 0.095 |
|  | Post-hoc | No significant differences | No significant differences |
| Frequency | F | F(3.967,345.101)=121.964 | F(4.007,348.591) =211.071 |
|  | p = | <0.001 | <0.001 |
|  | Post-hoc | t-test showed slopes < 0  t(89)=-17.86, p<0.001 | t-test showed slopes < 0  t(89)=-23.26, p<0.001 |
| Group * Frequency | F | F(7.933,345.101)=0.916 | F(8.014,348.591)=1.339 |
|  | p = | 0.502 | 0.223 |

*Type 2 submovements*

| *4 Hz* | *10 Hz* |
| --- | --- |
| *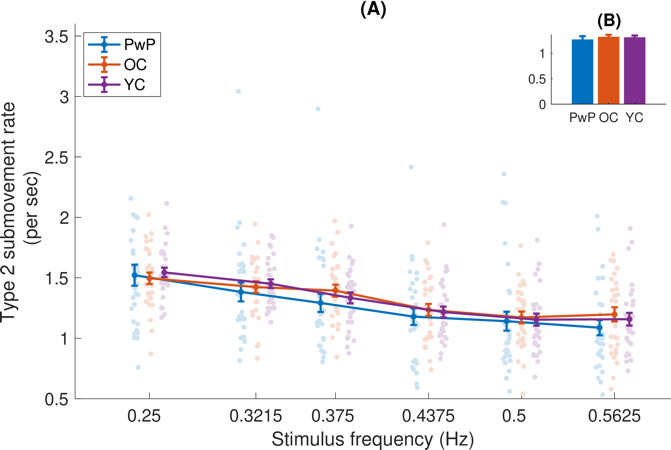* | 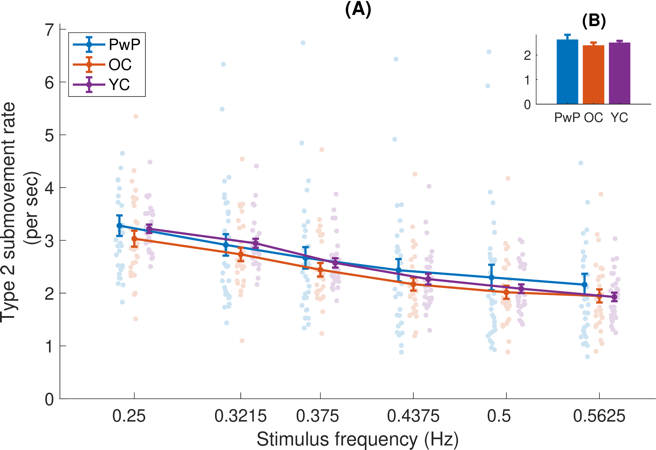 |

*Supplementary figure 3. Comparison of type 2 submovement rate with a 4 Hz and 10 Hz lowpass Butterworth filter.*

*Supplementary table 7. Results of a mixed-design ANOVA on the type 2 submovement rate, with both a 4 Hz and 10 Hz lowpass Butterworth filter.*

|  |  | 4 Hz | 10 Hz |
| --- | --- | --- | --- |
| Group | F | F(2,87)=0.272 | F(2,87)=0.643 |
|  | p = | 0.763 | 0.528 |
|  | Post-hoc | N/A | N/A |
| Frequency | F | F(3.510,305.409)=91.614 | F(4.074,354.442) =252.991 |
|  | p = | <0.001 | <0.001 |
|  | Post-hoc | t-test showed slopes < 0  t(89)=-14.29, p<0.001 | t-test showed slopes <0  t(89)=-25.70, p<0.001 |
| Group * Frequency | F | F(7.021,305.409)=1.017 | F(8.148,354.442) = 1.294 |
|  | p = | 0.419 | 0.244 |

*Type 3 submovements*

| *4 Hz* | *10 Hz* |
| --- | --- |
| *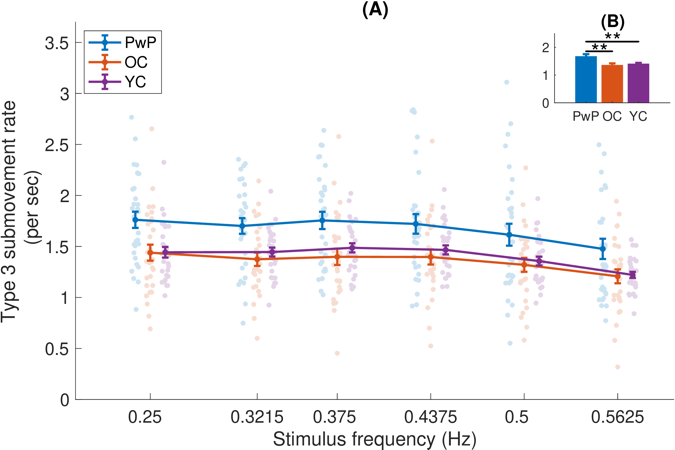* | 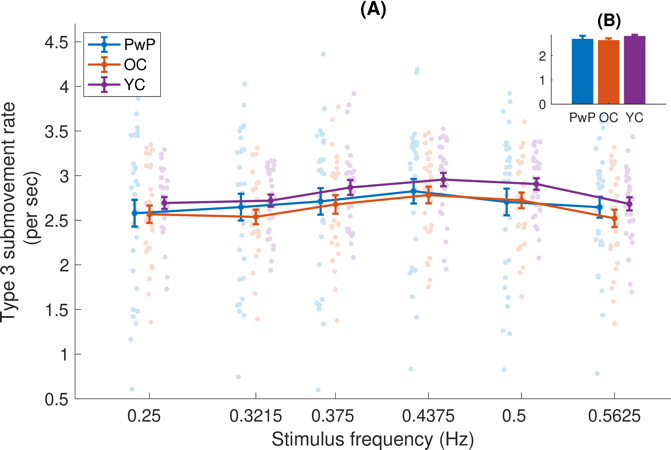 |

*Supplementary figure 4. Comparison of type 3 submovement rate with a 4 Hz and 10 Hz lowpass Butterworth filter. The inset highlights significant between-group differences.*

*Supplementary table 8. Results of a mixed-design ANOVA on the type 3 submovement rate, with both a 4 Hz and 10 Hz lowpass Butterworth filter.*

|  |  | 4 Hz | 10 Hz |
| --- | --- | --- | --- |
| Group | F | F(2,87)=6.751 | F(2,87)=0.744 |
|  | p = | 0.002 | 0.478 |
|  | Post-hoc | PwP > [OC (p=0.003), YC (p=0.009)] | N/A |
| Frequency | F | F(3.219,280.051)=25.949 | F(4.213,366.523)=15.465 |
|  | p = | <0.001 | <0.001 |
|  | Post-hoc | t-test showed slopes < 0  t(89)=-6.05, p<0.001 | t-test show trend < 0  t(89)=1.98, p=0.051 |
| Group * Frequency | F | F(6.438,280.051)=0.419 | F(8.426,366.523)=0.604 |
|  | p = | 0.878 | 0.783 |

**Supplementary results – analysis as a function of peak velocity**

For completeness, as we also varied peak velocity along with frequency, we also present the results as a function of peak velocity.

*Submovement rate (type 2+3 combined)*


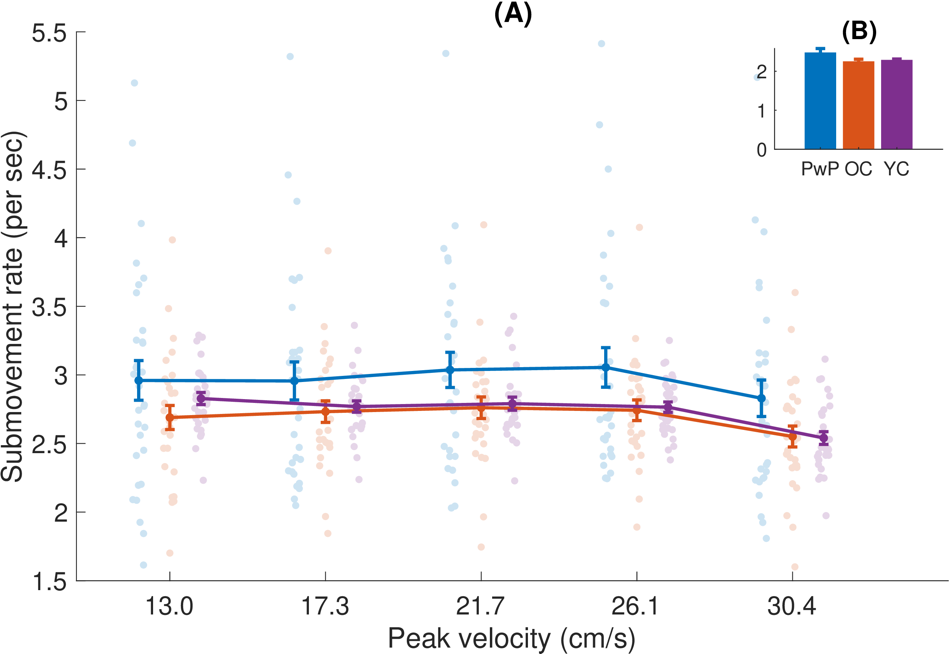


*Supplementary figure 5. Combined submovement rate (type 2 and 3) as a function of peak velocity. The inset highlights significant between-group differences.*

A main effect of group was not observed, although there was a trend (F(2,87)=2.510, p=0.087), however the post-hoc comparisons were not significant (all p>0.124). A main effect of peak velocity was observed (F(4,348)=26.829, p<0.001). The slopes of regression lines fit to each participant were significantly less than 0 (t(89)=-5.64, p<0.001, d=-0.59). An interaction was not observed (F(8,348)=1.598, p=0.124).

*Type 2 submovement rate*


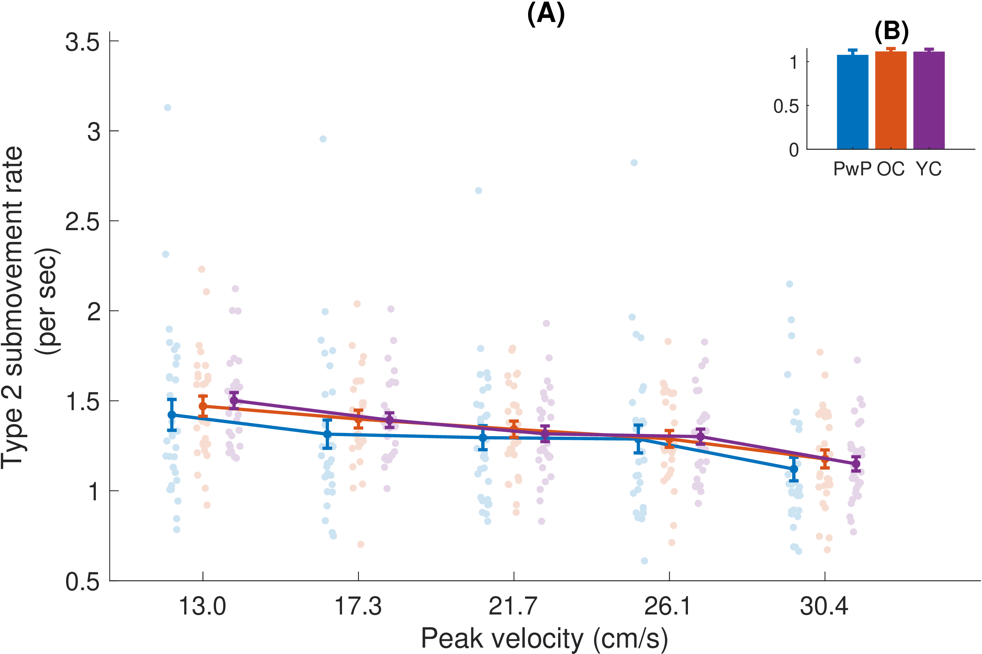


*Supplementary figure 6. Type 2 submovement rate as a function of peak velocity.*

A main effect of group was not observed (F(2,87)=0.242, p=0.785). A main effect of peak velocity was observed (F(3.423,297.806)=69.809, p<0.001). Regression fits of individual subject type 2 submovement rates showed that the slopes were negative (t(89)=-13.33, p<0.001, d=-1.39). An interaction of group and frequency was not observed (F(6.846,297.806)=0.747, p=0.630).

*Type 3 submovement rate*


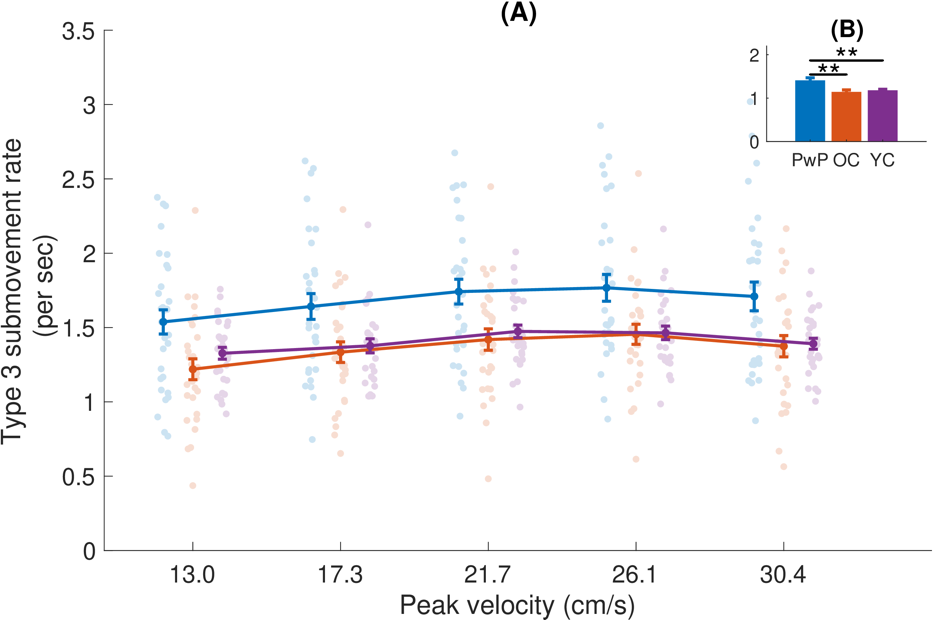


*Supplementary figure 7. Type 3 submovement as a function of peak velocity. The inset highlights significant between-group differences.*

A main effect of group was observed (F(2,87)=7.010, p=0.002). Post-hoc tests showed that the PwP group (1.679 ± 0.064) had significantly more type 3 submovements than both the OC group (1.360 ± 0.067, t(87)=3.440, p=0.003) and the YC group (1.407 ± 0.066, t(87)=2.967, p=0.008). A main effect of peak velocity was also observed (F(3.567,310.352)=26.319, p<0.001). Regression fits of individual subject type 3 submovement rates showed that the slopes were positive (t(89)=6.23, p<0.001, d=0.65). An interaction of group and peak velocity was not observed (F(7.135,310.352)=0.756, p=0.627).

*Position error – dX*

*
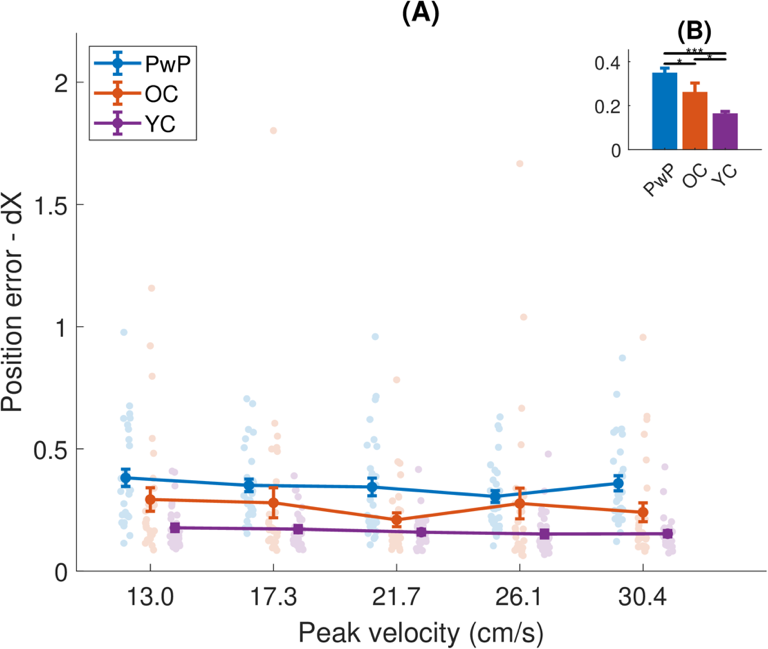
*

*Supplementary figure 8. Position error (dX) as a function of peak velocity. The inset highlights significant between-group differences.*

A main effect was observed for group (F(2,87)=11.152, p<0.001). Post-hoc tests showed that PwP had the highest dX (0.348 ± 0.028), which was significantly higher than the OC group (0.260 ± 0.028; t(58)=2.226, p=0.034, d=0.47), which in turn was significantly higher than the YC group (0.163 ± 0.028; t(57)=2.435, p=0.034, d=0.52). The effect of peak velocity was not significant although a trend was observed (F(3.385,294.490)=2.151, p=0.086), the interaction was also not significant (F(6.770,294.490)=1.044, p=0.400).

*Timing error - dT*


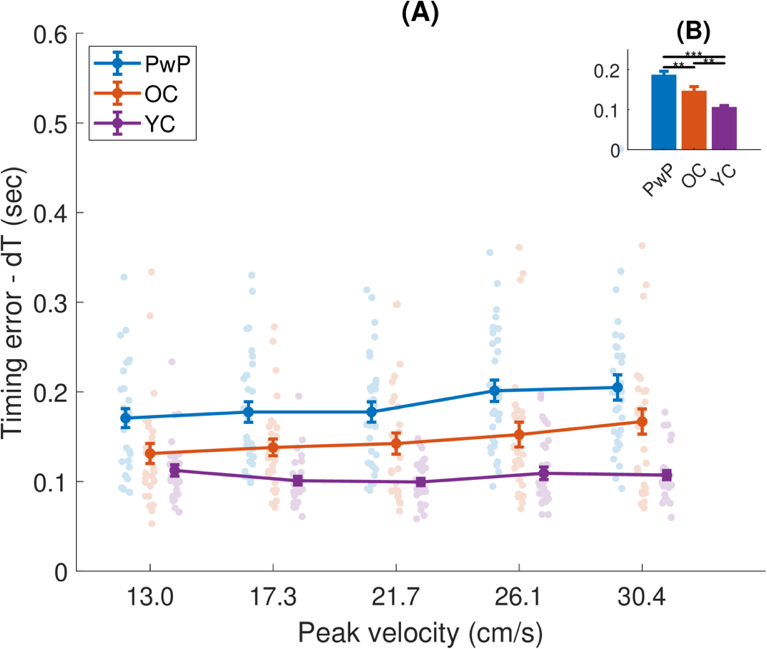


*Supplementary figure 8. Timing error (dT) as a function of peak velocity. The inset highlights significant between-group differences.*

A main effect of group was similarly observed for dT (F(2,87)=20.807, p<0.001). Post-hoc tests showed that PwP had the highest dT (0.186 ± 0.009), which was significantly higher than the OC group (0.146 ± 0.009; t(58)=3.193, p=0.004, d=0.71), which in turn was significantly higher than the YC group (0.106 ± 0.009; t(57)=3.177, p=0.004, d=0.71). A main effect of peak velocity was also observed (F(3.416,297.209)=8.746, p<0.001). Regression lines fit to individual subjects showed a positive slope (t(89)=4.29, p<0.001, d=0.45). In addition, an interaction of peak velocity and group was observed (F(6.832,297.209)=2.273, p=0.030). We similarly fit regression lines to each group individually, and found that the slope was positive for the PwP (t(30)=3.17, p=0.004) and OC (t(28)=3.79m p=0.001) groups, but not for the YC (t(29)=-0.16, p=0.876) group, which was not found to be significantly different from 0.

**Supplementary results – use of submovements per stroke rather than submovement rate**

In this analysis, rather than computing the number of submovements per second, we instead calculate the number of submovements per stroke. A stroke is defined here as a movement from left to right, or from right to left, i.e. a complete sine wave consists of two strokes. We note that in general, this will lead to an increase in the number of submovements (compared to the submovement rate per second) for the lower frequency movements (0.25 Hz, 0.3215 Hz, 0.375 Hz and 0.4375 Hz), as their duration is longer than 1 second, no change for 0.5 Hz (because the stroke duration is 1 second), and a decrease in the number of submovements for 0.5625 Hz.

For the combined number of submovements (consisting of both type 2 and type 3 submovements), see Supplementary Figure 9, we observed a trend for the effect of group (F(2,87)=2.638, p=0.077). Post-hoc tests did not show significant differences between the groups (all p>0.1). A main effect of frequency was also observed (F(2.336,203.205)=1999.144, p<0.001) – the number of submovements per stroke decreased as the frequency increased, as shown by negative slopes found using linear regression (i.e., t-tests showed they were less than zero: t(89)=-55.67, p<0.001, d=-5.82). A significant interaction of frequency and group was also observed (F(4.671,203.205)=3.232, p=0.009). Post-hoc tests showed differences between groups only at 0.25Hz between the PwP and OC groups (p=0.0325), and between the PwP and YC groups (p=0.0403).


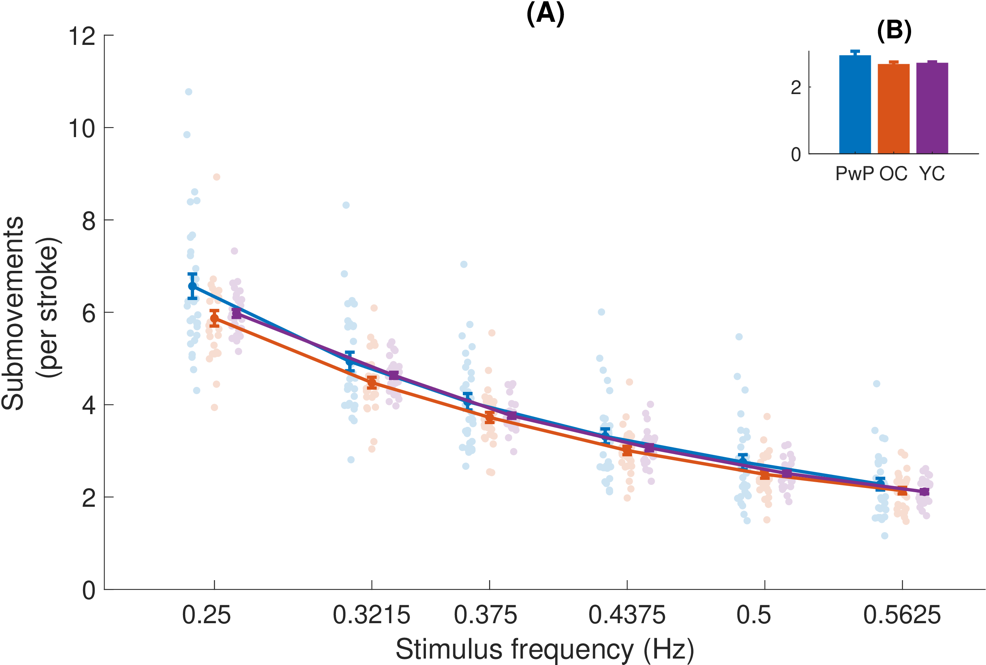


*Supplemental Figure 9. (A) Number of submovements per stroke (types 2 and 3 pooled together), as a function of frequency. Each dot is an individual participant, the dots are randomly shifted horizontally within a given frequency for clarity. (B) The inset shows the main effect of group (averaged across frequencies) - means and standard errors.*

For type 2 submovements, see Supplementary Figure 10, a main effect of group was not observed (F(2,87)=0.204, p=0.815). A main effect of frequency was observed (F(2.200,191.369)=909.883, p<0.001). As the frequency increased, the number of submovements decreased, as shown by negative slopes from linear regression fits to the data for each participant (t(89)=-37.31, p<0.001, d=-3.90). The interaction was not significant (F(4.399,191.369)=0.761, p=0.563).

**
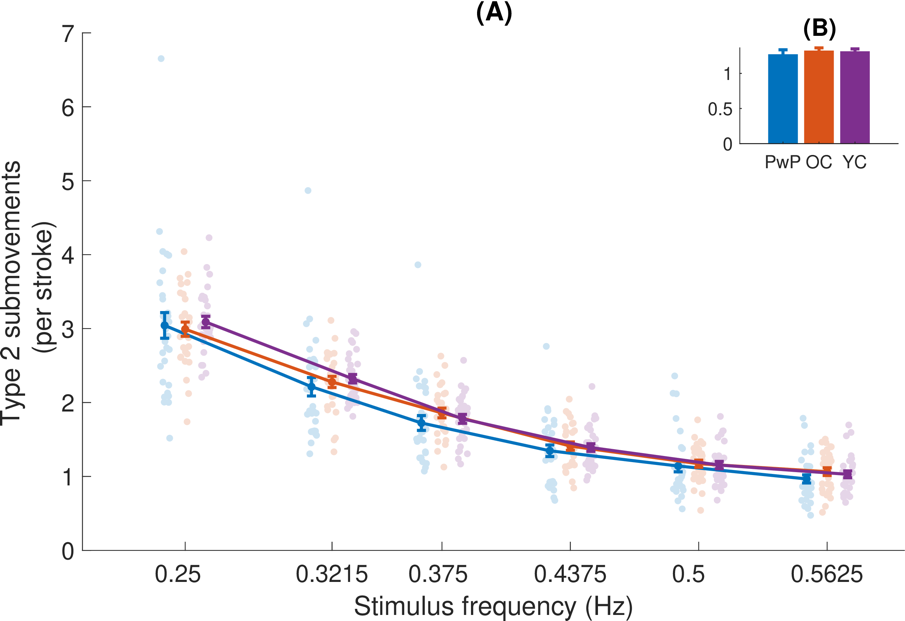
**

*Supplemental Figure 10. (A) Number of type 2 submovements per stroke, as a function of frequency. Each dot is an individual participant, the dots are randomly shifted horizontally within a given frequency for clarity. (B) The inset shows the main effect of group (averaged across frequencies) - means and standard errors.*

For type 3 submovements, see Supplementary Figure 11, a main effect of group was observed (F(2,87)=7.079, p=0.001). Post-hoc tests showed that the PwP group produced more submovements (2.247 ± 0.085) than both the OC group (1.822 ± 0.088; t(87)=3.457, p=0.003, d=0.79) and the YC group (1.883 ± 0.087; t(87)=2.983, p=0.007, d=0.68). Additionally, a main effect of frequency was observed (F(2.199, 191.299)=593.949, p<0.001). As before, this was due to a negative slope in a regression fit, as shown by t-tests of the slope which were significantly less than 0 (t(89)=-29.84, p<0.001, d=-3.12). An interaction was also observed for group and frequency (F(4.398,191.299)=3.232, p=0.011). Post-hoc tests only showed differences between groups at the lower frequencies and not at the higher frequencies (the following were significantly different: At 0.25 Hz: Between PwP and the YC; at 0.3215 Hz, between PwP and OC; the remainder are not significantly different).

**
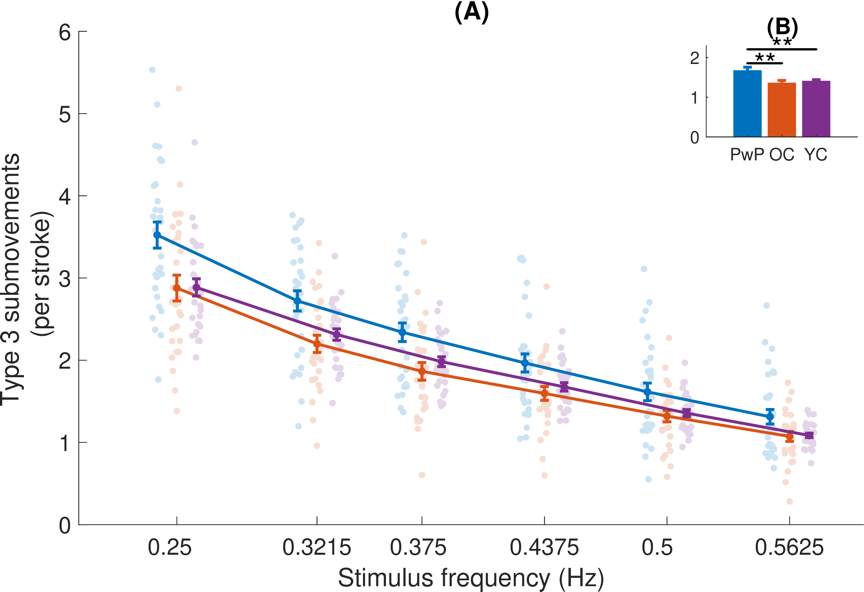
**

*Supplemental Figure 11. (A) Number of type 3 submovements per stroke, as a function of frequency. Each dot is an individual participant, the dots are randomly shifted horizontally within a given frequency for clarity. (B) The inset shows the main effect of group (averaged across frequencies) - means and standard errors, with significant differences shown by horizontal black lines. ** indicates significant differences at the level of p<0.01.*
